# Right on track? Performance of satellite telemetry in terrestrial wildlife research

**DOI:** 10.1371/journal.pone.0216223

**Published:** 2019-05-09

**Authors:** M. P. G. Hofman, M. W. Hayward, M. Heim, P. Marchand, C. M. Rolandsen, J. Mattisson, F. Urbano, M. Heurich, A. Mysterud, J. Melzheimer, N. Morellet, U. Voigt, B. L. Allen, B. Gehr, C. Rouco, W. Ullmann, Ø. Holand, N. H. Jørgensen, G. Steinheim, F. Cagnacci, M. Kroeschel, P. Kaczensky, B. Buuveibaatar, J. C. Payne, I. Palmegiani, K. Jerina, P. Kjellander, Ö. Johansson, S. LaPoint, R. Bayrakcismith, J. D. C. Linnell, M. Zaccaroni, M. L. S. Jorge, J. E. F. Oshima, A. Songhurst, C. Fischer, R. T. Mc Bride, J. J. Thompson, S. Streif, R. Sandfort, C. Bonenfant, M. Drouilly, M. Klapproth, D. Zinner, R. Yarnell, A. Stronza, L. Wilmott, E. Meisingset, M. Thaker, A. T. Vanak, S. Nicoloso, R. Graeber, S. Said, M. R. Boudreau, A. Devlin, R. Hoogesteijn, J. A. May-Junior, J. C. Nifong, J. Odden, H. B. Quigley, F. Tortato, D. M. Parker, A. Caso, J. Perrine, C. Tellaeche, F. Zieba, T. Zwijacz-Kozica, C. L. Appel, I. Axsom, W. T. Bean, B. Cristescu, S. Périquet, K. J. Teichman, S. Karpanty, A. Licoppe, V. Menges, K. Black, T. L. Scheppers, S. C. Schai-Braun, F. C. Azevedo, F. G. Lemos, A. Payne, L. H. Swanepoel, B. V. Weckworth, A. Berger, A. Bertassoni, G. McCulloch, P. Šustr, V. Athreya, D. Bockmuhl, J. Casaer, A. Ekori, D. Melovski, C. Richard-Hansen, D. van de Vyver, R. Reyna-Hurtado, E. Robardet, N. Selva, A. Sergiel, M. S. Farhadinia, P. Sunde, R. Portas, H. Ambarli, R. Berzins, P. M. Kappeler, G. K. Mann, L. Pyritz, C. Bissett, T. Grant, R. Steinmetz, L. Swedell, R. J. Welch, D. Armenteras, O. R. Bidder, T. M. González, A. Rosenblatt, S. Kachel, N. Balkenhol

**Affiliations:** 1 Wildlife Sciences, University of Goettingen, Goettingen, Germany; 2 School of Environment, Natural Resources and Geography, Bangor University, Bangor, United Kingdom; 3 Centre for African Conservation Ecology, Nelson Mandela University, Port Elizabeth, South Africa; 4 Norwegian Institute for Nature Research, Trondheim, Norway; 5 Office National de la Chasse et de la Faune Sauvage, Direction de la Recherche et de l'Expertise, Unité Ongulés Sauvages, Juvignac, France; 6 LECA, CNRS, Université Savoie Mont Blanc, Université Grenoble Alpes, Grenoble, France; 7 Freelance consultant, Milan, Italy; 8 Wildlife Ecology and Management, University of Freiburg, Freiburg, Germany; 9 Department of Conservation and Research, Bavarian Forest National Park, Grafenau, Germany; 10 Centre for Ecological and Evolutionary Synthesis, Department of Biosciences, University of Oslo, Oslo, Norway; 11 Leibniz Institute for Zoo and Wildlife Research, Berlin, Germany; 12 CEFS, Université de Toulouse, INRA, Castanet-Tolosan, France; 13 Institute for Terrestrial and Aquatic Wildlife Research, University of Veterinary Medicine, Hannover, Germany; 14 University of Southern Queensland, Institute for Agriculture and the Environment, Toowoomba, Queensland, Australia; 15 Department of Evolutionary Biology and Environmental Studies, University of Zurich, Zurich, Switzerland; 16 Centre d’Ecologie Fonctionnelle et Evolutive, UMR 5175, Centre National de la Recherche Scientifique (CNRS), Montpellier, France; 17 Department of Wildlife Ecology and Management, Landcare Research, Dunedin, New Zealand; 18 Department of Zoology, Facultad de Ciencias de la Universidad de Córdoba, Córdoba, Spain; 19 University of Potsdam, Potsdam, Germany; 20 Leibniz Centre for Agricultural Landscape Research (ZALF), Müncheberg, Germany; 21 Department of Animal and Aquacultural Sciences, Norwegian University of Life Sciences, Ås, Norway; 22 Department of Biodiversity and Molecular Ecology, Fondazione Edmund Mach, San Michele all'Adige, Italy; 23 Forest Research Institute of Baden-Wuerttemberg, Freiburg, Germany; 24 Research Institute of Wildlife Ecology, University of Veterinary Medicine, Vienna, Austria; 25 Wildlife Conservation Society, Mongolia Program, Ulaanbaatar, Mongolia; 26 University of Ljubljana, Biotechnical Faculty, Department for Forestry, Ljubljana, Slovenia; 27 Grimsö Wildlife Research Station, Department of Ecology, Swedish University for Agricultural Sciences (SLU), Riddarhyttan, Sweden; 28 Snow Leopard Trust, Seattle, United States of America; 29 Department of Migration and Immuno-ecology, Max-Planck Institute for Ornithology, Radolfzell, Germany; 30 Lamont-Doherty Earth Observatory, Columbia University, Palisades, New York, United States of America; 31 Panthera, New York, NY, United States of America; 32 Department of Biology, University of Florence, Florence, Italy; 33 Vanderbilt University, Department of Earth & Environmental Sciences, Nashville, TN, United States of America; 34 Programa de Pós-Graduação em Zoologia, Laboratório de Ecologia Espacial e Conservação, Instituto de Biociências, Departamento de Ecologia, Universidade Estadual Paulista (UNESP), Rio Claro, Brasil; 35 Ecoexist, Maun, Botswana; 36 University of Oxford, Oxford, United Kingdom; 37 Texas A&M University, College Station, Texas, United States of America; 38 Haute ecole du paysage, d'ingenierie et d'architecture de Geneve, Genève, Switzerland; 39 Faro Maro Ecoresearch, Departamento de Boquerón, Paraguay; 40 Guyra Paraguay—CONACYT, Asunción, Paraguay; 41 Instituto Saite, Asunción, Paraguay; 42 The Ronin Institute, Montclair, NJ, United States of America; 43 Institute of Wildlife Biology and Game Management, University of Natural Resources and Applied Life Sciences, Vienna, Austria; 44 Centre National de la Recherche Scientifique, Lyon, France; 45 Laboratoire de Biométrie et Biologie Évolutive, UMR CNRS 5558, Université Claude Bernard Lyon 1, Villeurbanne cedex, France; 46 Institute for Communities and Wildlife in Africa, Department of Biological Sciences, University of Cape Town, Cape Town, Western Cape, South Africa; 47 Cognitive Ethology Laboratory, German Primate Center (DPZ), Leibniz Institute for Primate Research, Goettingen, Germany; 48 School of Animal, Rural and Environmental Sciences, Nottingham Trent University, Brackenhurst Campus, Southwell, United Kingdom; 49 Office of Environment and Heritage, Wollongong, NSW, Australia; 50 Division of Forestry and Forest Resources, Norwegian Institute of Bioeconomy Research, Ås, Norway; 51 Centre for Ecological Sciences, Indian Institute of Science, Bangalore, India; 52 Ashoka Trust for Research in Ecology and the Environment, New Dehli, India; 53 Wellcome Trust/DBT India Alliance, Hyderabad, India; 54 School of Life Sciences, University of KwaZulu-Natal, Durban, South Africa; 55 D.R.E.Am. Italia, Pratovecchio Stia, Italy; 56 Office National de la Chasse et de la Faune Sauvage, Unités Ongulés Sauvages, Birieux, France; 57 Trent University, Peterborough, Ontario, Canada; 58 SUNY College of Environmental Science and Forestry, Syracuse, NY, United States of America; 59 University of Santa Catarina, Florianópolis, Santa Catarina, Brazil; 60 Onçafari, Pinheiros -São Paulo, Brazil; 61 Wetlands and Coastal Ecology Branch, US Army Engineer Research and Development Center, Environmental Laboratory, Vicksburg, MS, United States of America; 62 Wildlife and Reserve Management Research Group, Department of Zoology and Entomology, Rhodes University, Grahamstown, South Africa; 63 School of Biology and Environmental Sciences, University of Mpumalanga, Nelspruit, South Africa; 64 Ministery of Environment and Natural Resources of Mexico, Mexico City, Mexico; 65 Alianza Nacional Para la Conservacion del Jaguar, A.C. Mexico, Mexico; 66 Biological Sciences Department, California Polytechnic State University, San Luis Obispo, California, United States of America; 67 Facultad de Ciencias Agrarias, Universidad Nacional de Jujuy–CONICET, San Salvador de Jujuy, Argentina; 68 Tatra National Park, Zakopane, Poland; 69 Humboldt State University, Arcata, California, United States of America; 70 The Cape Leopard Trust, South Africa, Cape Town, Western Cape, South Africa; 71 Centre National de la Recherche Scientifique HERD Program Hwange LTER, Main Camp Research, Hwange National Park, Hwange, Zimbabwe; 72 University of British Columbia, Vancouver, British Columbia, Canada; 73 Department of Fish and Wildlife Conservation, Virginia Tech, Blacksburg, VA, United States of America; 74 Département d'étude du milieu naturel et agricole, Service public de Wallonie, Gembloux, Belgium; 75 Research Institute for Nature and Forest, Brussels, Belgium; 76 Departamento de Ciências Biológicas, Instituto de Biotecnologia, Universidade Federal de Goiás/Regional Catalão (UFG), Catalão, Goiás, Brazil; 77 Programa de Conservação Mamíferos do Cerrado (PCMC), Fazenda Limoeiro, Cumari, Goiás, Brazil; 78 Department of Zoology, University of Venda, Thohoyandou, South Africa; 79 Instituto de Pesquisa e Conservação de Tamanduás no Brasil, Parnaíba, Piauí, Brazil; 80 Global Change Research Institute CAS, Department of Biodiversity Research, Brno, Czech Republic; 81 Wildlife Conservation Society—India, Bangalore, Karnataka, India; 82 University of Applied Sciences and Arts of Western Switzerland, Delémont, Switzerland; 83 Macedonian Ecological Society, Skopje, Macedonia; 84 Office National de la Chasse et de la Faune Sauvage, Kourou, France; 85 UMR EcoFoG (AgroParisTech, Cirad, CNRS, INRA, Université des Antilles, Université de Guyane), Kourou, French Guiana; 86 Departamento de Conservación de la Biodiversidad, El Colegio de la Frontera Sur, Campeche, Mexico; 87 Agence Nationale de Sécurité Sanitaire de l'Alimentation, de l'Environnement et du Travail | ANSES · Nancy Laboratory for Rabies and Wildlife, Nancy, France; 88 Institute of Nature Conservation, Polish Academy of Sciences, Krakow, Poland; 89 Wildlife Conservation Research Unit, Department of Zoology, University of Oxford, Oxford, United Kingdom; 90 Department of Bioscience—Wildlife Ecology, Aarhus University, Aarhus, Denmark; 91 Department of Wildlife Ecology and Management, Faculty of Forestry, Duzce University, Düzce, Turkey; 92 Behavioral Ecology and Sociobiology Unit, German Primate Center (DPZ), Leibniz Institute for Primate Research, Goettingen, Germany; 93 University of Goettingen, Goettingen, Germany; 94 Department of Zoology and Entomology, Rhodes University, Grahamstown, South Africa; 95 Courant Research Centre “Evolution of Social Behaviour”, University of Goettingen, Goettingen, Germany; 96 WWF Thailand, Bangkok, Thailand; 97 Queens College, City University of New York, New York, NY, United States of America; 98 University of Cape Town, Cape Town, South Africa; 99 Grupo de Investigación en Ecología del Paisaje y Modelación de Ecosistemas-ECOLMOD, Departamento de Biología, Facultad de Ciencias, Universidad Nacional de Colombia, Bogotá, Colombia; 100 Department of Environmental Science, Policy and Management, University of California, Berkeley, California, United States of America; 101 University of North Florida, Jacksonville, FL, United States of America; 102 School of Environmental and Forest Sciences, University of Washington, Seattle, Washington, United States of America; University of Lleida, SPAIN

## Abstract

Satellite telemetry is an increasingly utilized technology in wildlife research, and current devices can track individual animal movements at unprecedented spatial and temporal resolutions. However, as we enter the golden age of satellite telemetry, we need an in-depth understanding of the main technological, species-specific and environmental factors that determine the success and failure of satellite tracking devices across species and habitats. Here, we assess the relative influence of such factors on the ability of satellite telemetry units to provide the expected amount and quality of data by analyzing data from over 3,000 devices deployed on 62 terrestrial species in 167 projects worldwide. We evaluate the success rate in obtaining GPS fixes as well as in transferring these fixes to the user and we evaluate failure rates. Average fix success and data transfer rates were high and were generally better predicted by species and unit characteristics, while environmental characteristics influenced the variability of performance. However, 48% of the unit deployments ended prematurely, half of them due to technical failure. Nonetheless, this study shows that the performance of satellite telemetry applications has shown improvements over time, and based on our findings, we provide further recommendations for both users and manufacturers.

## Introduction

Wildlife telemetry units equipped with satellite functionality offer an attractive set of functions for remotely tracking individual animal movements across a large diversity of species [1,2]. Modern satellite telemetry devices allow for tracking movements at unprecedented temporal and spatial scales, yielding large amounts of detailed information. Since the early 1990s, geolocation satellite tags, largely relying on the Global Positioning System (GPS) satellite network, have been used successfully to locate animals in wildlife research for a variety of purposes [3,4], including the study of predator-prey interactions [5], foraging behavior [6], activity patterns [7], movement patterns [8], migratory routes [9], habitat preferences [10], and other aspects of animal behavior [11,12]. The scope of applications in research and conservation continues to increase due to the addition of accelerometers, gyroscopes, magnetometers, cameras and environmental sensors, as well as improvements in technology (e.g. increased battery life, solar charging, increased memory storage) leading to significant reductions in the size and weight of devices [2,13]. Wildlife satellite tracking is producing ‘big data’ and has been suggested as a means to monitor environmental changes [2,14]. Its success is exemplified by the increasing number of scientific studies published (see S1 Fig;[15]), the development of collaborative e-infrastructures to aid with the management, analysis and sharing of large movement data sets (e.g. Movebank, Eurodeer, WRAM, see also [16]), and new data analysis methods being presented in the literature at rapid pace [17–19]. Indeed, Kays et al. claimed that we are at the start of the golden age of animal tracking science [2].

Against this backdrop of rapid development, it is crucially important to understand the relative influence of various factors (e.g. environmental characteristics of the study area, species traits, unit specifications, satellite constellation) on the performance of satellite telemetry devices across species and habitats. Such factors can considerably reduce data volume and quality [3,20–22], and their influence needs to be understood to identify where further improvements can be made. Additionally, acquiring a sufficient number of devices to conduct a scientifically robust project usually involves considerable financial investment and is logistically demanding, while equipping animals with satellite telemetry devices requires proper expertise and careful ethical consideration with respect to animal welfare. Hence, it is of paramount importance to maximize the effectiveness of any deployed units.

**Fig 1:**
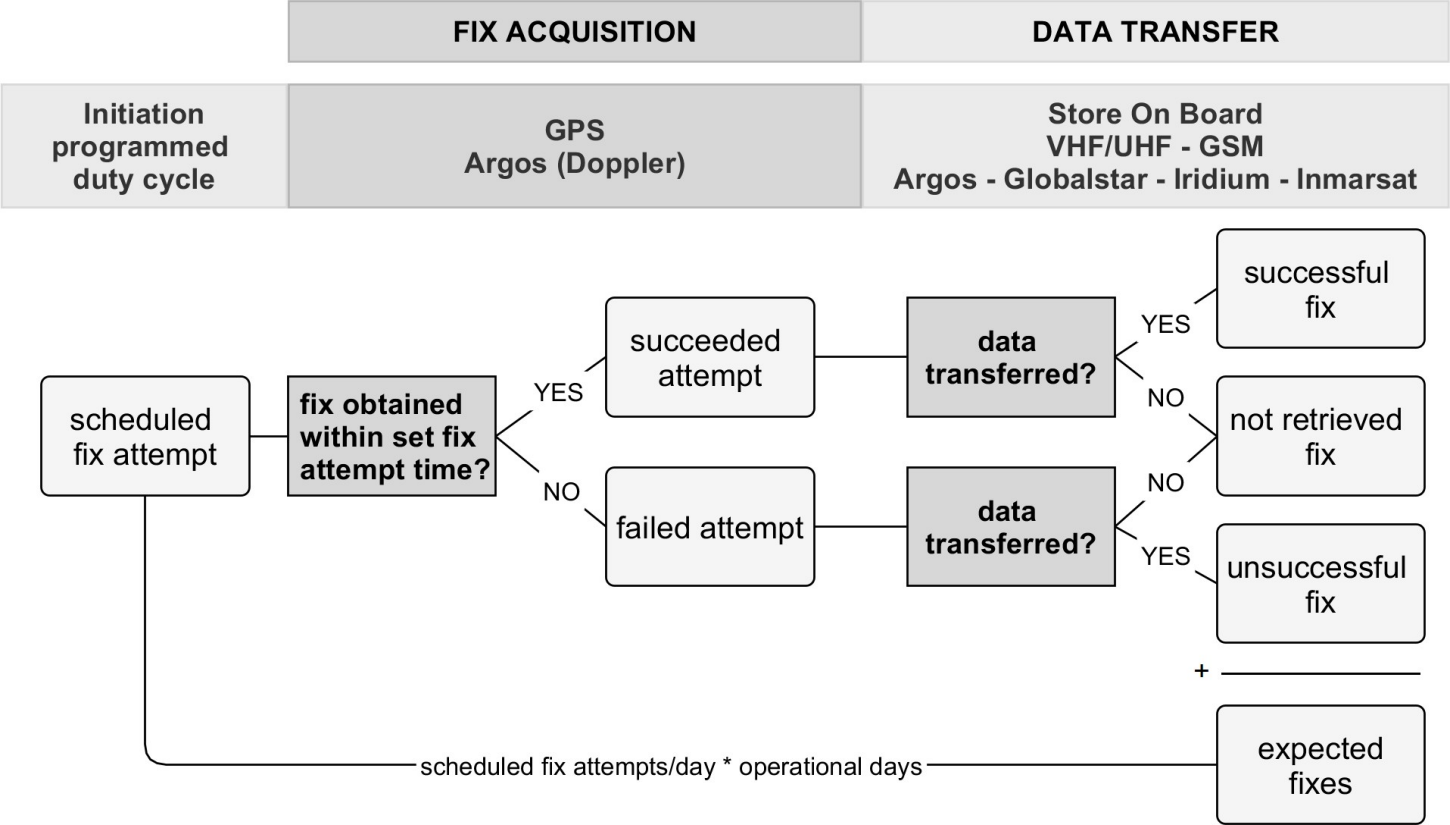
Two-step satellite telemetry process. The general two-step operation of terrestrial satellite telemetry units, and the possible fix outcomes of the process. The number of expected fixes equals the sum of successful, unsuccessful and not-retrieved fixes (see Materials and methods).

In its simplest form, a satellite telemetry unit is a uniquely identifiable bio-logging tag consisting of a satellite geolocation sensor in conjunction with the necessary components to process, store and retrieve the geolocation data [23]. The general operation of satellite telemetry units involves two main steps (Fig 1). The first step is *fix acquisition*: whereby according to a pre-programmed (usually cyclic) schedule, the geolocation satellite tag scans the sky for satellites, attempts to calculate the coordinates of its position on Earth, and records its coordinates for that exact time (a process often referred to as ‘obtaining a fix’). The second step is retrieving the data from the device or *data transfer*: whereby the results stored on the unit (i.e., unit locations and potentially additional information such as accelerometer data) are transferred to the user.

Fix acquisition nowadays is almost exclusively done using the Global Positioning System (GPS) and numerous studies have investigated the factors affecting the efficiency of satellite telemetry devices in acquiring fixes, which include unit brand and antenna orientation [24,25], fix attempt interval and frequency [26,27], species behavior [11,28], percent canopy cover [29,30], vegetation cover type [31], satellite constellation [32], topography [33] and in urban settings the density and height of buildings [34]. These factors can cause high variation in the volume and quality (i.e. geographic accuracy) of fixes stored on the unit and can introduce bias in the geographical spread of the obtained locations [35].

In contrast to research focusing on fix acquisition, far fewer studies have addressed the success in transferring data from the unit to the user (e.g. [36]; see also S1 Table), despite the fact that the data transfer rate can considerably impact the amount of data eventually available to the user, especially in cases where the chances of recovering the unit from the field are small [37]. The choice of data transfer method depends on the biology of study species, environmental characteristics of the study area [3], practical considerations (e.g. battery life, project budget) and previous experiences, as well as the study objectives.

In addition to data loss during the two-step telemetry process, data are also lost when units suffer premature technical failures (e.g. wear, production errors, memory storage or battery malfunction, etc.), when the animal dies (e.g. natural causes, hunting or collision with vehicles), or when animals succeed in removing the unit [21,22,37]. Despite the availability of a number of data screening and processing techniques to deal with biased and missing locational data [35,38,39], reduced data quality and volume can prevent units from providing adequate data to answer specific research questions that were formulated based on the premise that greater amounts of high-quality data would be available. These limitations have led some authors to urge for caution when opting for satellite telemetry in wildlife research [20].

The multitude of factors affecting the performance of satellite telemetry devices makes it challenging to evaluate their efficiency in wildlife research. To our knowledge, few large-scale evaluations of the overall efficiency of units across species and habitats have been conducted (e.g. [22]; see also S1 Table), and we currently do not have a thorough understanding of how reliable units are for providing the expected large volume of high-quality data under various circumstances. Moreover, projects where the quantity or quality of gathered information is inadequate are unlikely to produce any peer-reviewed publications and thus we expect the literature to be biased towards successful applications [22]. For example, Campbell et al. [40] report that approximately 50% of studies involving satellite transmitters in Australasia remained unpublished, partially due to insufficient data quality or quantity. Hence, an evaluation based on literature review alone would likely lead to biased conclusions. Here, we use a questionnaire approach to assess the performance of satellite telemetry units in terrestrial wildlife research across the globe. Our specific objectives were to investigate the (i) fix acquisition success, (ii) data transfer success, (iii) overall success rate of units in providing fixes to the user (the combination of i and ii), and the proportion of deployments ending prematurely due to (iv) technical malfunctioning or (v) other causes (see Materials and Methods). In addition, we evaluate how the success rates are impacted by environmental, species and unit characteristics.

## Materials and methods

### Data collection

To avoid a potential bias in published literature towards successful satellite telemetry studies, we used a standardized questionnaire format (see S1 Questionnaire). We first used a short online form explaining the study, in which we invited researchers to submit their contact details if they were interested in participating. We shared the form on conservation and ecology related mailing lists, online wildlife and technology platforms, and via social networks for scientists. We also mailed the invitation to contribute to personal contacts, authors of satellite telemetry studies, and contributors to online animal movement databases. We then sent the standardized data gathering questionnaire to those who had shown interest in participating. The questionnaire was approved by the Ethics Committee of the College of Natural Sciences at Bangor University (Ethical consent number: cns2015mh1).

The design of the questionnaire was guided by the general operation of satellite telemetry (see Fig 1). In the two-step process of data collection in satellite telemetry, the first step is fix acquisition. Nearly all recent, commercially available units for wildlife research use the GPS satellite network for the purpose of obtaining fixes, although other methods exist (e.g. Argos with Doppler-effect, Global Navigation Satellite System or GLONASS for short, see [3, 4]). The geolocation tag picks up the radio signals from a number of satellites, calculates its distance from each satellite, and then uses an algorithm to determine its position [23]. Each programmed fix attempt by the unit either succeeds or fails in obtaining a fix, and this result is stored on an inbuilt memory device, the capacity and reliability of which are usually not limiting the quantity of data acquired. The tag needs an unobstructed line-of-sight to at least four satellites to obtain a reliable 3D fix, with fewer satellites leading to lower spatial accuracy of the obtained coordinates [27,41]. The programmed fix attempt frequency itself can influence the success of the fix attempts: if the time lag between fix attempts is short, the unit can reuse the satellites’ ephemeris data, reducing the time and battery power needed to determine the next location, and thus increasing the likelihood of success [26,27,42]. Additionally, factors such as the spatial distribution of satellites in the sky and the distortion of the satellites’ radio signals due to atmospheric effects can introduce imprecision in the obtained positions [41]. This imprecision is measured as the geometric dilution of precision (DOP). The number of available satellites and the DOP for each fix attempt are usually—but not always—included in the fix result information provided to the user by the unit or the manufacturer. The second step in data collection is the transfer of the obtained locations from the unit to the user. Several options for transferring data are available [3,4], including:

- The store-on-board unit is retrieved from the field through recapturing animals or after the unit drops off, and the user extracts the data using a physical connection between the unit and a computer.
- A VHF or UHF transmitter on the unit enables the user to download the data remotely, typically using a hand-held receiver from a relatively short distance. This method can be used if animals can be approached closely enough and resources are available to visit the area frequently.
- The unit is equipped with a GSM component and SMS messages containing the fix results are sent to a server or sometimes directly to the user’s cellular phone. Although GSM coverage is widespread, some wildlife species tend to roam in remote areas where coverage is low (e.g. [43]).
- An additional satellite tag on the unit transmits the fix results to a commercial communication satellite network usually providing global or near-global coverage (e.g. Argos, GlobalStar, Iridium, Inmarsat).

Where the additional unit mass is not of concern, satellite telemetry units can be equipped with an automated timed-release mechanism to avoid having to recapture the animal to retrieve the collar. Additionally, the units usually include traditional VHF transmitters for on-the-ground triangulation or homing-in, as well as activity and environmental sensors that provide supplementary information [2]. This supplementary information is not usually included in remote data transfer to reduce the size of the individual data packages to be transferred.

### Standardized questionnaire

The questionnaire consisted of 27 questions, grouped according to the topic they addressed: study area and animal characteristics, unit specifications, deployment details, unit costs, and researcher opinion on data quality and quantity and the usefulness for their career, for conservation, and for research. In line with Fig 1, we considered the number of *expected fixes* to be the number of scheduled fix attempts that were initiated by the unit between the day of deployment and the moment the unit failed, the animal died or until the last data download. A fix was considered *successful* when a scheduled fix attempt succeeded in obtaining the unit’s location *and* the user subsequently managed to retrieve the time and coordinate information for this fix from the unit. We considered a fix to be *unsuccessful* when a scheduled fix attempt failed in obtaining the unit’s location, but the user still managed to subsequently retrieve the available information for this attempt from the unit. Note that the available information for unsuccessful fixes includes variables such as date, time and number of visible satellites, but lacks coordinates. Fixes with obviously improbable coordinates were also counted as unsuccessful. Any scheduled fix attempt for which no information was transferred to the user—hence where it is unknown whether the unit succeeded or failed to obtain its location—was considered a *not-retrieved fix*. We neglected the quality of individual fixes (DOP, 2D/3D) for the purposes of this study, because the diversity of ways to measure the quality would have increased the complexity of our questionnaire disproportionately (see Recommendations). Although failure of memory devices themselves may hinder data acquisition, the specific issue was not raised in any of the contributions to this study or similar studies [22], indicating that the capacity and reliability of recent memory devices used in terrestrial satellite tracking applications are usually not limiting. We did not consider this factor in our questionnaire.

### Unit performance and its covariates

We compiled a set of covariates that potentially influence the different measures of unit success. Several of these covariates were derived directly from the questionnaire, while area-based covariates were calculated from study area coordinates (see Table 1).

**Table 1:** Boosted beta regression covariates.

Name	Description	Type	Level
Brand	The manufacturer of the majority of units in the project	Qualitative	Unit
No. of units	The number of units deployed in the project	Quantitative	Unit
Purchase date	Weighted mean of the year of purchase of all units in the project	Quantitative	Unit
Time-to-fix	Weighted mean of the maximum time units were allowed to obtain a fix	Quantitative	Unit
Transfer method	The transfer method used by the majority of units in the project. Levels: GSM; Satellite; Store-on-board; VHF/UHF.	Qualitative	Unit
Burrowing/ Hibernating	Boolean indication of burrowing and/or hibernating individuals in the project	Qualitative	Species
Height (log-transformed)	Natural log of the weighted mean of the species height across all individuals in the project	Quantitative	Species
Forest Cover (quantitative)	Mean forest cover in the study area as derived from the GlobCover dataset using the coordinates provided in the questionnaire.	Quantitative	Environment
Forest cover (qualitative)	Percentage of forest cover as indicated in the questionnaire. Levels: 0–25%; 26–50%; 51–75%; 76–100%	Qualitative	Environment
Forest type	Type of forest in the study area as indicated in the questionnaire. Levels: No forest cover; Temperate evergreen; Temperate deciduous; Temperate mixed; (Sub)Tropical evergreen; (Sub)Tropical deciduous; (Sub)Tropical mixed.	Qualitative	Environment
Forest density	Density of forest in the study area as indicated in the questionnaire. Levels: No forest cover; Open understory & sparse canopy cover; Dense understory & sparse canopy cover; Open understory & intermediate canopy cover; Dense understory & inter-mediate canopy cover, Open understory & closed canopy; Dense understory & closed canopy.	Qualitative	Environment
Terrain ruggedness	Terrain ruggedness as indicated in the questionnaire. Levels: Steep slopes and narrow valleys, flat areas and gentle slopes are rare (< 20%); Steep slopes, interspersed with flat areas and/or gentle slopes; Mostly flat area and/or gentle slopes, with occasional steep slopes (< 20%); Mostly flat area or gentle slopes (< 5% steep slopes).	Qualitative	Topography
Terrain Ruggedness Index	Mean Terrain Ruggedness Index across the study area, as derived from either SRTM or ASTER Digital Elevation Models for the study area. This variable was used as a proxy for available view to the sky.	Quantitative	Topography

Covariates used for the boosted beta regression on the fix acquisition rate and overall fix success rate of satellite telemetry units.

We used five measures to evaluate unit success. Three measured the success during different parts of the actual satellite telemetry data gathering process (the success rates), while the other two measured the rate of failures in deployments (failure rates).

#### Fix acquisition rate

The fix acquisition rate measures the proportion of transferred fix attempts that was successful, regardless of the number of originally scheduled fix attempts. It was calculated as *successful / (successful + unsuccessful) fixes* (see Fig 1).

#### Data transfer rate

To evaluate the data transfer success, we calculated the data transfer rate as *(successful + unsuccessful fixes) / expected fixes* (see Fig 1). To compare the data transfer success of different transfer methods (GSM, VHF/UHF, satellite), we excluded projects where additional fixes were downloaded from units with remote data transfer after recovering them from the field: these additional fixes were not transferred using the remote transfer functionality of the unit.

#### Overall fix success rate

The overall fix success rate reflects the proportion of scheduled fix attempts that succeeded in obtaining a fix, *and* for which information was subsequently successfully transferred to the user, either remotely or by physical connection. In contrast to the fix acquisition rate, it accounts for data loss during both the fix acquisition and the data transfer processes and evaluates the proportion of scheduled fixes that were eventually available to the user. This also includes fixes that were downloaded from the unit after retrieval from the field and therefore the Overall fix success rate is also influenced by the number of retrieved units. We calculated it as *successful / expected fixes* (see Fig 1). In previous research, the overall fix success rate is often referred to as the fix success rate or FSR [34,37,44], whereby authors have either assured or assumed complete data transfer.

#### Deployment failure rate

The proportion of deployments that ended prematurely for any given reason (animal-related, technical or unknown) is calculated as the deployment failure rate.

#### Technical failure rate

The proportion of deployments ending prematurely exclusively due to known technical issues (including failure of timed-release mechanism) is called the technical failure rate and is a subset of the deployment failure rate.

### Statistical analysis

All analyses were conducted in R version 3.4.3 [45]. To evaluate the relative importance of covariates for the fix success and overall fix success rates, we used boosted beta regression models [46]. The boosted beta regression approach combines the beta regression framework, as a special case of the Generalized Additive Models for Location, Scale and Shape (GAMLSS) class of regression models [47,48], with the gradient boosting framework. Beta regression is a commonly used technique in the natural sciences to model a continuous response bounded between 0 and 1, with essentially the same interpretation as logistic regression [46,49]. The flexibility of the beta distribution allows for complex responses to be modelled, while the GAMLSS class allows precise model specification as it enables the fitting of not only the conditional mean, but also other parameters of the distribution of the response variable (location, scale, and shape) as a function of explanatory variables and/or random effects [48]. Additionally, where classical beta regression commonly uses maximum likelihood to optimize regression coefficients and requires the user to select variables based on model-selection criteria (e.g. AIC; [50]), the boosted beta regression approach uses an algorithm called gamboostLSS [47]. The algorithm uses the gradient boosting framework to optimize the models for all distribution parameters (see [46,51]). The family of beta distributions, as implemented in the R package gamboostLSS [51–53], has two parameters: *μ* is the conditional mean, while *φ* is the precision or overdispersion. The conditional variance of the outcome is then given as *μ(1-μ)/(1+φ)*. Essentially, boosted beta regression assesses the relative importance of covariates simultaneously on the mean (*μ*) and on the variability (in terms of overdispersion *φ*) of the dependent variable by iteratively fitting simple regression functions of the effects of each covariate to the negative gradient for *μ* and *φ*. Relative variable importance for the models of both parameters is given as the percentage of boosting iterations in which a covariate was selected as the best fit to the respective parameter of the outcome. When the algorithm is appropriately tuned, e.g. via cross validation, it has the major added advantage of having an in-built mechanism for variable selection [46,52]. We applied this approach in our study using the R package gamboostLSS version 1.2–1 [51]. All covariates described above were entered in the overall success model (S2 Code), while the main data transfer method variable was excluded for the fix acquisition rate model (S1 Code). We log-transformed the species height variable and included the main brand used in each project as a random variable. Variable distributions are presented in S2 Fig. We weighted each project by the number of telemetry units deployed in it.

## Results

**Fig 2:**
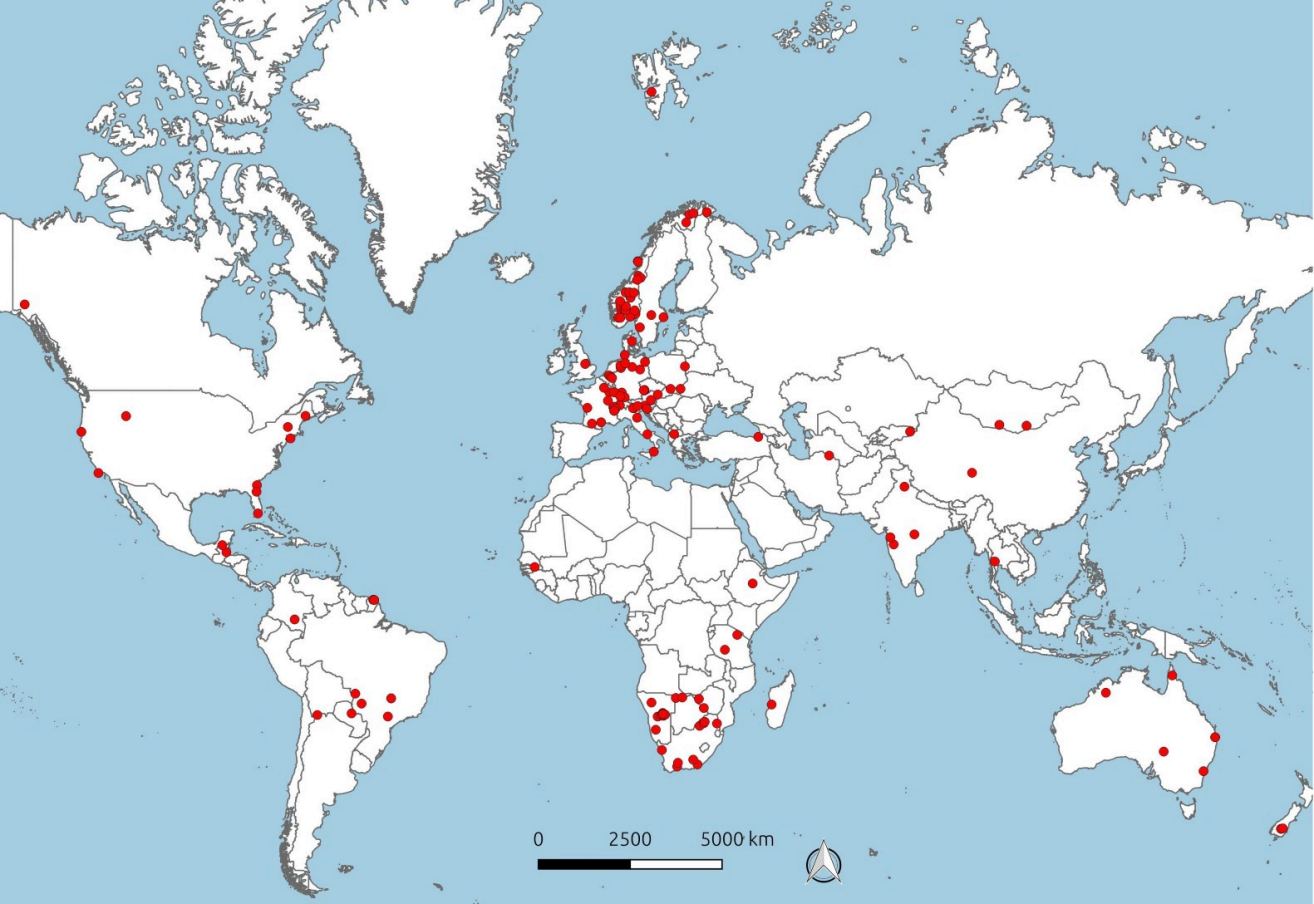
Project distribution. The geographic distribution of all projects that provided information on the performance of satellite telemetry units. Note that each red dot can comprise more than one project.

We combined information from 167 projects in 142 study areas across 42 countries and 6 continents (see Fig 2). The geographic distribution was uneven, with just over half of all study areas located in Europe, 20% in Africa and less than 10% in each of the other continents. Projects ran between 2001 and 2017 and lasted on average 3.5 years, ranging between 60 days and 14.3 years. Across all projects, a total of 3,695 individuals of 62 terrestrial wildlife species were equipped with 3,130 telemetry units of 16 brands. Most units were purchased between 2006 and 2015. Reptiles and (ground-dwelling) birds were tagged in four and two study areas respectively, whereas small to large mammals were the study subjects in all other areas (see S2 Table). An analysis of all trends in the observed data is presented in S1 and S2 Texts.

### Overall unit performance

In the average project, 93.6% (range: 23.4–100%) of the fix attempt information was transferred successfully from the units to the user, and 85.2% (7.5–100%) of these transferred fix attempts had succeeded in calculating the unit’s position (i.e. obtaining a fix). Eventually, users obtained on average 77.5% (6.7–100%) of the fixes that they could have expected to obtain during the total time that units were deployed and functioning properly in the project. However, 25.2% (0–100%) of all unit deployments in an average project ended prematurely due to technical failure, irrespective of the unit’s price or purchase date. Additionally, about as many deployments ended prematurely due to animal-related issues (e.g. mortality, unit removal) or for unknown reasons, such that 47.5% (0–100%) of all unit deployments in an average project ended earlier than was planned.

### Fix acquisition rate

**Fig 3:**
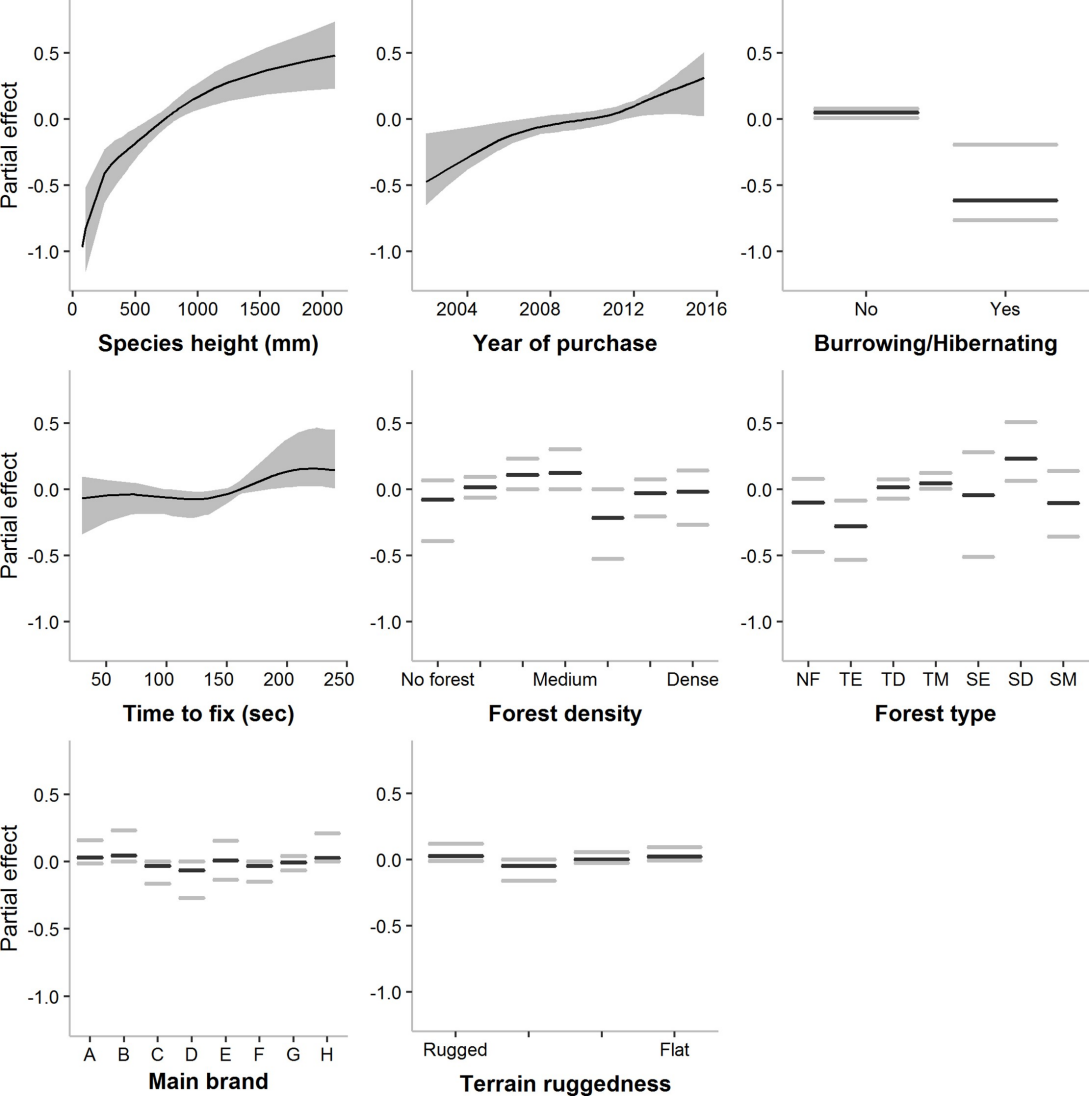
Covariate partial effects on the mean Fix acquisition rate. Mean-centered partial effects of the most important variables predicting the mean (*μ*) fix acquisition rate of satellite telemetry units (empirical confidence intervals in grey). Graphs are presented left-to-right in order of importance. Partial effects display the effect of the variable while accounting for all other variables in the model. Forest type levels: NF = No forest cover; TE = Temperate evergreen; TD = Temperate deciduous; TM = Temperate mixed; SE = (Sub)Tropical evergreen; SD = (Sub)Tropical deciduous; SM = (Sub)Tropical mixed.

The boosted beta regression model selected species height followed by the purchase date of the units as the most important variables determining the mean fix acquisition rate (Table 2). The model predicted the fix acquisition success to be higher for taller species, with the effect leveling off with increasing height. Fix acquisition rate was also higher for more recently purchased units, and for units with a longer maximum allowed time-to-fix (see Fig 3). Burrowing or hibernating behavior reduced the fix acquisition rate. The main brand used in a project and the qualitative environmental variables (forest type, forest density and terrain ruggedness) were selected in less than 15% of the boosting iterations and lacked a consistent trend. However, the variability in fix acquisition rate was influenced strongly by the main brand used and increased in dense forest environments and in intermediate to highly rugged terrain (see Table 2 and S3 Fig). In contrast, a longer allowed time-to-fix decreased the variability.

**Table 2:** Selection frequencies of covariates for both the mean (*μ*) and variability (*φ*) parameters in the boosted beta regression model for the fix acquisition rate.

Variable	Selection frequency	
	*μ*	*φ*
Height	22%	
Purchase date	19%	
Burrowing/Hibernating	15%	
Time to fix	11%	20%
Forest density	11%	30%
Forest type	11%	
Brand	7%	40%
Terrain ruggedness (qualitative)	4%	
Terrain Ruggedness Index		10%

The higher the selection frequency, the more important the covariate is in predicting either the mean or the variability of the fix acquisition rate.

### Data transfer rate

In 11.6% of all projects (N = 167), multiple data transfer methods were used simultaneously. In these cases, the main data transfer method per project was determined by simple majority. In 15% of all projects, the majority of units were store-on-board units, where the data transfer was entirely dependent upon the successful retrieval of the units from the field. All remaining projects primarily used remote data download methods to transfer the information from the units to the user. GSM was the main data transfer method in 40.1% of all projects, while 23.1% mainly used satellite communication and 21.8% mainly VHF/UHF.

**Fig 4:**
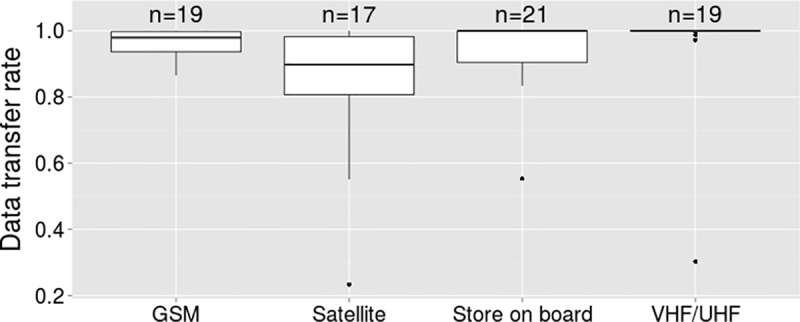
Data transfer success. Data transfer rate per main transfer method used in the projects.

Note that the data transfer rate is based on data that were transferred remotely *and* data that were downloaded from retrieved units. In 64 out of 125 projects in which data were transferred remotely, retrieved units provided additional fixes to the remotely transferred fixes. To compare data transfer success between transfer methods, we needed to exclude those projects to isolate the effects of the data retrieval method on the overall success rate. Thus, we selected only projects where either all fixes were transferred via a physical connection (store-on-board units), or all fixes were obtained through remote data download (see Materials and Methods). Due to the reduced number of projects, some factor levels had insufficient sample sizes for a boosted beta regression approach and we could not determine the relative importance of different factors affecting the transfer rate for each transfer method. However, the observed trend in the data indicated that data transfer success using communication satellite systems was lowest and most variable, while VHF/UHF was most effective in data transfer (see Fig 4).

### Overall fix success rate

**Fig 5:**
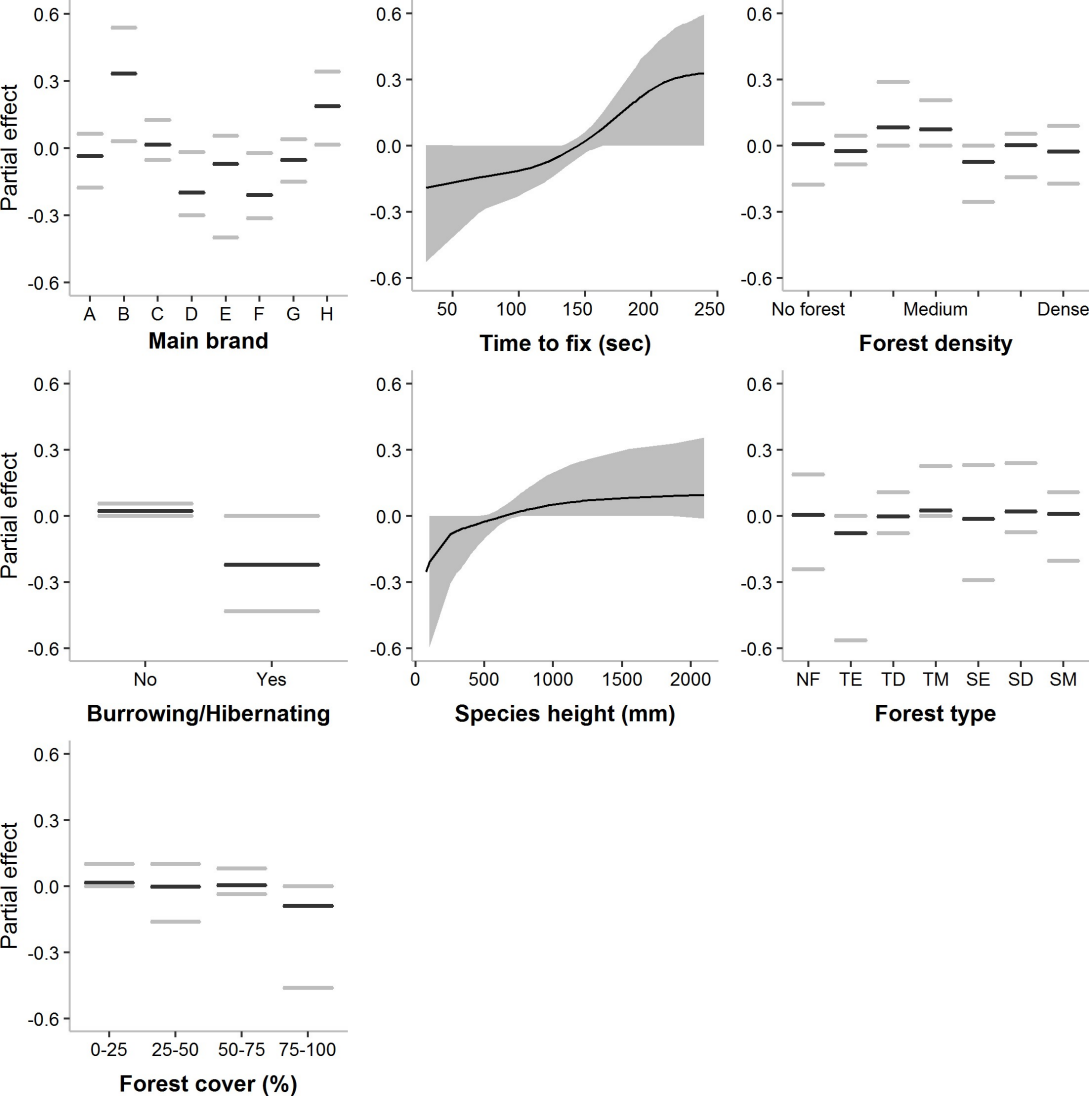
Covariate partial effects on the mean Overall fix success rate. Mean-centered partial effects of the most important variables predicting the mean (*μ*) overall fix success rate of satellite telemetry units (empirical confidence intervals in grey). Graphs are presented left-to-right in order of importance. Partial effects display the effect of the variable while accounting for all other variables in the model. Forest type levels: NF = No forest cover; TE = Temperate evergreen; TD = Temperate deciduous; TM = Temperate mixed; SE = (Sub)Tropical evergreen; SD = (Sub)Tropical deciduous; SM = (Sub)Tropical mixed.

The overall fix success rate model was the same as the fix acquisition rate model, but with the overall fix success rate as the dependent variable and the main data retrieval method added as an explanatory variable. As a random factor, the main brand had the strongest effect on mean overall success rate followed by the maximum allowed time-to-fix, while species and vegetation characteristics were less important (Table 3). As with the fix acquisition rate, units allowing a longer time-to-fix were predicted to yield higher overall fix success, as did taller, non-burrowing or non-hibernating species. Forest density did not show a consistent effect, whereas a forest cover of over 75%, especially temperate evergreen forest, seemed to slightly reduce the overall fix success rate (Fig 5). The variability of the overall fix success rate was mostly determined by the unit brand and environmental characteristics (Table 3). Intermediate levels of forest cover and a higher mean Terrain Ruggedness Index increased the variability of the overall fix success rate, while forest type and density did not show consistent effects (S4 Fig). Variability was reduced in projects with more recent units and with units allowing a longer time-to-fix. Of all projects that reported an overall fix success rate (n = 144), 11.81% had an overall fix success rate below 50%.

**Table 3:** Selection frequencies of covariates for both the mean (*μ*) and variability (*φ*) parameters in the boosted beta regression model for the overall fix success rate.

Variable	Selection frequency	
	*μ*	*φ*
Brand	43%	20%
Time-to-fix	19%	10%
Height	10%	
Burrowing/Hibernating	10%	
Forest Density	10%	10%
Forest type	5%	20%
Forest cover (qualitative)	5%	
Forest cover (GlobCov)		20%
Purchase date		10%
Terrain Ruggedness Index		10%

The higher the selection frequency, the more important the covariate is in predicting either the mean or the variability of the fix acquisition rate.

### Failure rates

**Fig 6:**
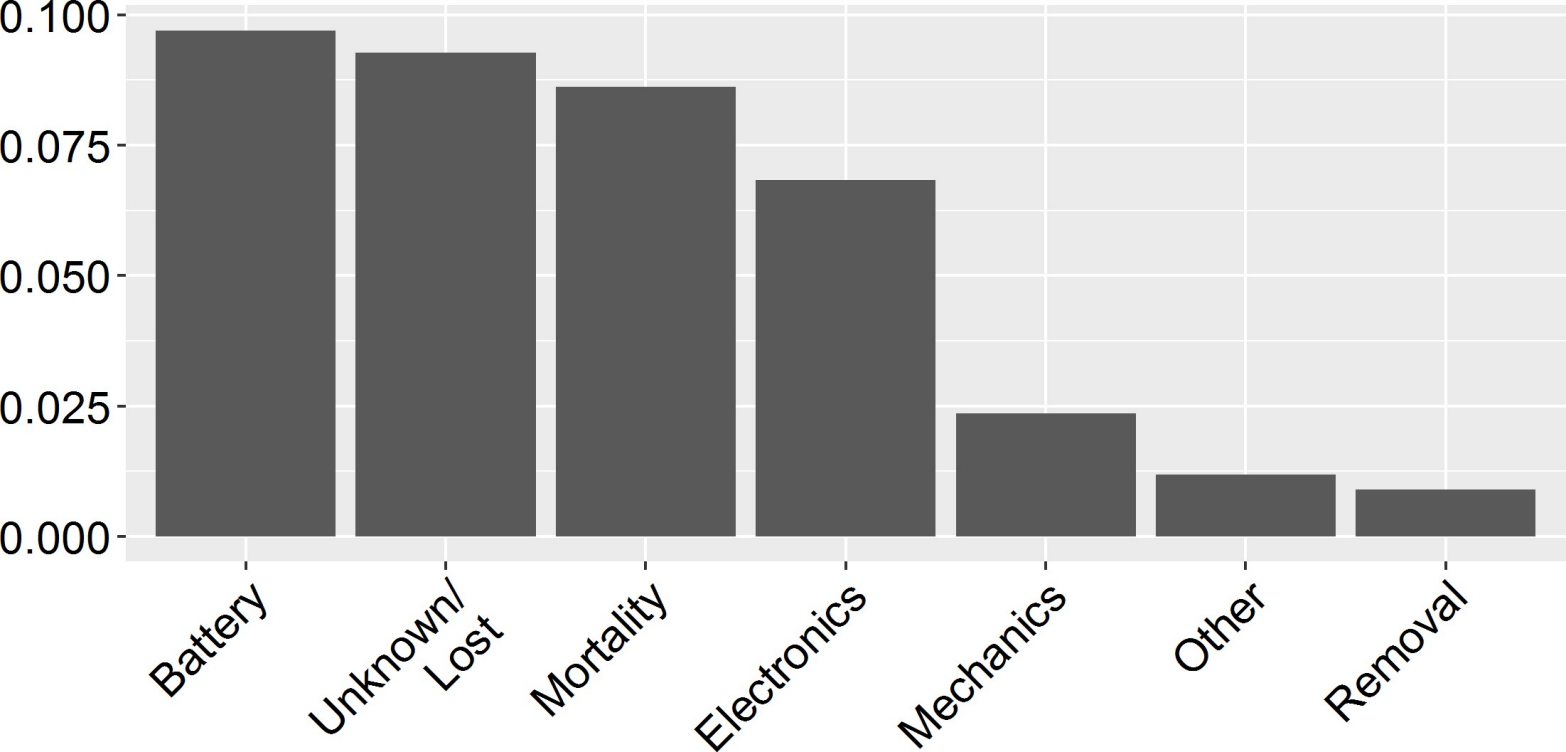
Causes for premature ending of deployments. Proportion of reported deployments ending prematurely due to various unit or animal-related factors.

Of all unit deployments for which the cause of termination was reported (n = 2,124), 61.2% were either successfully ongoing or ended as planned, while 18.9% ended due to technical malfunctioning. Technical malfunctions (n = 401) were due to battery failures (51.4%), electronic (36.2%) or mechanical problems (12.5%). Approximately 1 out of 10 unit deployments (10.5%) ended for unknown or non-specified reasons, and a similar amount ended due to either animal mortality (8.6%) or unit removal by the animal (0.9%; see Fig 6). Three out of the 2,214 deployments involved units that were equipped with solar-powered batteries and thus theoretically less likely to experience premature power loss. Of all projects that reported the deployment failure rate (n = 123), 38.2% had a failure rate of over 50%.

### Scientific outputs

Some terrestrial wildlife projects publish a considerable number of scientific papers and good examples of collaborative cross-project publications exists [54–56]. However, our results indicate that the scientific output for the projects in this study was generally low. Of all the projects that reported on scientific output (n = 68), 62.9% declared that no peer-reviewed papers had been published from the obtained data, while 17.2% published just one paper. However, 80.9% of the studies reported that they were gathering additional data or waiting for external factors to allow for publication. We also found that the data loss resulting from low success rates and high unit failure rates sometimes led to reduced scientific output of the project: 10.3% of the researchers indicated the lack of sufficient data quantity or quality as a reason for the low number of publications arising from the study. Almost half of all projects produced at least one publication that was not peer-reviewed (47.8%). Organizing, managing and sharing information is increasingly done online through e-infrastructures, the most well-known example of which is arguably Movebank. Of all 167 projects in our study, 112 (67%) reported on whether their data were uploaded to Movebank. Of these, 79.5% did not upload their data, primarily because researchers did not know of this online data system (36.4%) or were not familiar with its uses (23.4%).

## Discussion

In the global context of the rise of satellite tracking in wildlife research, there is a need to carefully evaluate the technique globally across species and habitats. Our analyses revealed that the average performance of satellite telemetry in terrestrial wildlife research has improved over the last 15 years, but still presents considerable opportunities for improvements, notably in fix acquisition and failure rates. We found that performance is more strongly influenced by unit and species characteristics than environmental conditions in a study area, but that environmental conditions increased the variability, influencing the technique’s effectiveness in various ways.

### Fix acquisition rate

Our average fix acquisition rate (85%) was above average in comparison with the range of 46–99% reported by Frair et al. [35] for a series of studies conducted between 2001 and 2010, and with the average of 66% reported by Matthews et al. [22] across 24 Australian studies on several mammal species between 2005 and 2011. However, the range in fix acquisition rate in our study was 7.5–100%, showing that the variability of fix acquisition rates generally remains very high. Our boosted beta regression analysis suggested that fix acquisition rate is highest for taller-standing species and recently purchased units with a long time-to-fix. Probably due to improved sensor technology and increased GPS satellite coverage, fix acquisition rate was higher in more recent telemetry units. For example, in 2011, the GPS network was expanded from a 24 to a 27-slot satellite constellation, which improved coverage in most parts of the world [57]. An increased fix acquisition rate for taller species intuitively makes sense because the units’ satellite view is generally less obstructed by understory vegetation (e.g. [58]). Additionally, the larger size of units for tall species allows for larger antenna structures, which improves unit performance. Generally, environmental variables (e.g. forest type, density, cover, and terrain ruggedness) do not seem to consistently influence the fix acquisition rate in a specific direction. While a longer maximum allowed time-to-fix and younger units were predicted to reduce variability, high forest density and terrain ruggedness were predicted to increase the variability of the fix acquisition rate in an area, indicating that under these circumstances, increasing the fix attempt frequency or number of units to account for expected data loss may be a good strategy.

### Data transfer rate

Not all fix attempt information that was successfully stored on the unit, was successfully transferred to the user. We could not directly compare data transfer rates between transfer methods, but the observed trend was that data transfer was most effective in VHF/UHF units. Store-on-board units and GSM units also performed well, whereas data transfer over communication satellite systems had the lowest success rate and was most variable. Argos, Iridium, GlobalStar and Inmarsat all claim global or near global coverage (Inmarsat and GlobalStar do not cover polar regions, and GlobalStar’s coverage in sub-Saharan Africa only began in 2015), but satellite-based data transfer suffers from the same limitations as geolocation satellite-based fix acquisition and can be expected to vary as a result.

However, we are aware that study site access and species mobility characteristics may be the main criteria influencing the choice and success of data retrieval system. In contrast to satellite-based data transfer, researchers can more accurately evaluate and control the chances of data transfer success using UHF and GSM, based on the presence of GSM sending towers and knowledge of species behavior.

### Overall fix success rate

Overall fix success rate was 78% across projects, meaning that studies should account for receiving 22% fewer fixes than expected under a given fix acquisition frequency. Imperfect overall success rates were generally due to issues with fix acquisition and unit failure, although in specific situations (e.g. dense evergreen forest) data transfer may be the more important reason.

### Failure rates

Lost and failing units cause additional data loss beyond the loss occurring during the two-step telemetry phase. In the average project, close to half of all units stopped working properly sooner than expected (including animal-related failures), and about half of these (i.e. 25.2% of all units) suffered a technical failure. In the early 2000s, Johnson et al. [20] reported a failure rate of 69.2%, of which all were suspected to be due to technical issues, while Gau et al. [37], Hebblewhite et al. [59], and Matthews et al. [22] experienced an overall deployment failure rate of 53.3%, 47.0%, and 47.6% respectively, more comparable to the average in this study. These numbers, as well as the observed trends as described in Fig 1.8 in S1 Text, suggest that failure rates have improved over the last decade, but remain an important potential source of data loss. Matthews et al. [22] also found that in 24% of all collars with timed-release mechanism (drop-off), the mechanism failed to release the unit on the scheduled time, or at all. In the present study, 10.5% of all unit deployments ended for unknown reasons (i.e. the units could not be retrieved from the field and were indeed ‘lost’). The average of collars reported as lost by Matthews et al. [22] was 10%, while Gau et al. [37] reported 18.3% lost collars. Any number of failing or lost units can result in considerable loss of data and investment [21,22,37], and there are often concerns that lost units may cause harm to individuals if the unit does not drop off automatically.

Some studies reported instances where telemetry units performed fix attempts much more frequently than programmed in the schedule, resulting in a much higher number of fixes than expected, and consequently a much shorter battery lifespan. Matthews et al. [22] also report this, as well as units failing to store fix results during (part of) the deployment period. It is unclear though whether the reported underperformance was due to environmental obstruction, limited storage capacity, data storage failure or data transfer issues. In our study, where fix rates were investigated on a project level, we were not able to quantify the number of units affected by such unintended shifts in duty cycle. Similarly, some projects involved flexible fix acquisition schedules whereby fix frequencies varied depending on the activity level of the animal or the crossing of a ‘virtual fence’ in the landscape. We included only those projects where the number of expected fixes could be accurately calculated or estimated.

### Study limitations

We used a questionnaire to obtain information on the performance of satellite telemetry devices, which had the clear advantage of avoiding a potential publication bias towards successful studies. However, there are also some caveats to the approach. Firstly, the geographic distribution of projects in our data set was biased towards European studies. We attribute this distribution to the willingness to participate and the communication reach of the study’s primary authors (e.g. limited information from Asia and Latin America due to language barriers). We recognize that the relatively low number of studies reported from North America in this paper is not an accurate representation of the work that has been conducted in the region, and we are aware of many published papers using satellite telemetry for wildlife research by universities and government agencies in North America [10,25,29,59–63]. This geographically uneven distribution of studies might have influenced the representation of different brands and our findings on the publication output of wildlife satellite telemetry studies. Similarly, the inclusion of more studies in heavily forested regions (e.g. the Amazon) or very steep areas (e.g. the Alps) could influence the importance of environmental parameters on the mean and the variability of the success rates.

Secondly, the data used for the overall fix success rate model included projects where data were downloaded both remotely and from retrieved units. The additional fixes downloaded after retrieval can significantly add to the overall fix success rate of a project. This means that the overall fix success rate was determined not just by the combined effect of the fix acquisition rate and the data transfer success, but also by the number of retrieved units. However, separating the fixes transferred remotely from those downloaded after retrieval was not possible because most researchers were not able to distinguish between these fixes in their final datasets.

Finally, even though many researchers may appreciate some insight on which manufacturer to buy from for a specific study, our study was not designed to evaluate the relative performance of different brands of satellite devices. For a number of reasons, we cannot draw reliable conclusions about the performance of individual brands. For example, we determined the main brand of a project on the basis of simple majority, meaning that the use of multiple brands within one project (which was the case in 12% of all projects) could confound the brand-specific effect. Also, the sample size (i.e. number of projects) for many brands was too low for any statistically valid comparisons. This is especially important because different brands were also used with varying frequency across years, and we found a substantial improvement of success rates with increasing year of purchase. Thus, instead of incorrectly treating main brand as an actual covariate, we chose to include it as a random variable in our boosted beta regression models. Our main goal was not to compare brands, but to identify environmental, species and technical variables that determine the units’ success rates. As a random factor, the main brand influenced the average fix acquisition and overall success rates (selection frequency of 7% and 43%, respectively), but also had the strongest impact on the variability in success rates (40% and 20% selection frequency for fix acquisition and overall success rates, respectively). In other words, predictions of mean success rates for each brand were associated with high uncertainties. This unpredictability could come, for example, from the rate of uptake of new technological advances throughout the years by different brands, or the deployment of the same brand on species of different sizes across years. Overall, we cannot give meaningful recommendations for choosing specific brands. Nevertheless, we can derive several important recommendations from our results, both for researchers wishing to deploy satellite telemetry devices, and for manufacturers of such devices.

## Recommendations

The scope of possibilities and the detail of information that can be obtained from satellite telemetry are major advantages of the technology for answering a range of ecological and conservation questions. However, given the considerable investment and the variability of the effects that many aspects of study design have on the success rate, we recommend carefully considering project objectives, study design, and budget constraints before investing in satellite telemetry units [3,20]. Specifically, we present important considerations to guide potential users in deciding if and which satellite telemetry units are useful to deploy in future studies. For manufacturers, we recommend ways to actively contribute to the improvement of satellite telemetry applications in wildlife research.

### Recommendations for users

#### Plan for more units than necessary

We found that 10% of all units were lost, while close to 20% suffered technical failures. When planning a project, we suggest budgeting for 10–20% more units or animals than strictly necessary for the study, in accordance with previous suggestions [22].

#### Use a higher-than-necessary fix rate

The average fix acquisition rate was 85%, while the overall success rate was 78%. Whenever possible (i.e. when not restricted by study species size and thus battery size), we recommend setting the fix frequency 15–25% higher than strictly necessary for the study design. This will not compensate for data loss due to animal mortality or unit failures but can counteract data loss that originates from species behavior, unit orientation or environmental factors temporarily obstructing satellite view. Additionally, if the study design allows the fix interval to be sufficiently short, the fix acquisition rate could be increased [26,27,42]. In order to save battery, a valuable approach could be to adopt a dynamic fix attempt schedule where the fix frequency is adjusted depending on the animal’s activity levels (as indicated by an accelerometer) or geographic location [13]. When using high-frequency or dynamic schedules, it is important to be aware of their effects on future analysis, e.g. autocorrelation, trajectory regularity, etc. [64,65]. For example, for many types of analyses, having trajectories with consistent fix intervals is as important, if not more so, than having trajectories with a high temporal resolution.

#### Report specifications and settings in publications

Satellite telemetry users should report unit specifications and settings as well as fix acquisition and data transfer rates in scientific publications or their supplementary materials. This will facilitate future comparisons between species, environments, and settings for different research questions (e.g. home-range, dispersal, connectivity, etc.). Developing a fully-fledged metadata reporting standard is outside the scope of this paper but would be very useful. The development of such a protocol should be a collaborative effort between users, manufacturers and e-infrastructures.

#### Upload data and meta-data

Additionally, researchers could use existing collaborative e-infrastructures to store and manage their data (e.g. Movebank, Eurodeer, and others; see [2,16]). Importantly, meta-data on unit specifications and settings as described above should be included in the upload, in as far as the database design allows for it. Online animal movement databases usually have options to let users decide if, when and how contributed data can be accessed by third parties.

### Recommendations for manufacturers

#### Standardize data transferred to user

We encourage manufacturing companies to collaborate with scientists and practitioners to develop minimum standards for the data type and format that is stored on the units and/or transferred to users. For example, some brands only provide a record of successful fixes to the user, and do not inform the user about the fate of the remaining fix attempts. However, it is useful for the user to know whether the remaining fix attempts failed during fix acquisition or data transfer in order to evaluate the suitability of either the unit settings or the data transfer method in a given study area [22]. Another example is the variety of ways that the precision of the fix is measured. Many manufacturers provide data on the number of satellites used for the fix, or the fix dimension (2D/3D). Some provide data on Positional Dilution of Precision (PDOP), while others only report on the horizontal component of PDOP (HDOP). Sometimes only horizontal error estimates are reported based on undisclosed proprietary algorithms. Producing a standard reporting format for data originating from animal-borne devices would be beneficial for the management and analysis of such data in collaborative e-infrastructures (e.g. Movebank, Eurodeer), and would increase the efficacy of satellite telemetry for large-scale studies [16,40]. Additionally, it would increase the feasibility of reviews such as this one. This standard reporting should streamline the data types and units, and variable names and definitions according to a common vocabulary [16]. The use of data standards has been endorsed by the International Biologging Society (https://www.bio-logging.net/).

#### Focus on reducing technical failure rates

A unit’s reliability is at least as important as its offer of additional functionality or superior performance. While animal mortality or tag removal by the animal are hard to eliminate as a cause for premature termination, reducing unit or data loss by avoiding technical failures (including drop-off mechanism) would potentially present a considerable increase in the final data volume obtained. Thus, trying to improve the technical reliability of satellite telemetry units in obtaining successful fixes and transferring them to users, as well as ensuring that units withstand environmental and animal-related impacts should be a major focus for manufacturers. Here, it may be noted that sometimes a unit with more basic but well-tested components is valued more than a unit with latest cutting-edge technology but increased uncertainty about performance. To help reduce failure rates, we encourage manufacturers to actively work with researchers on an easy-to-use and standardized feedback mechanism for users to report success and failure rates. In cases where the units cannot be sent back for diagnostics, knowing the circumstances of the failures could help identify the underlying cause.

#### Improve fix acquisition rate

While data transfer generally is less of an issue (except for satellite-based data transfer), the average fix acquisition rate of 85% in our study leaves scope for improvement. In the last decade, commercial satellite applications (e.g. hand-held GPS devices and smartphones) have begun using both the GPS and its Russian counterpart, GLONASS, while some already provide compatibility with Galileo, the European counterpart that is set to reach operational completion by2020. The addition of the GLONASS and Galileo networks nearly triples the number of potential satellites available for geolocation, which results in faster acquisition of more precise locations [66]. Using chipsets that provide access to multiple systems could increase fix success rates in challenging settings. Another opportunity is the recent development of miniaturized gyroscopes, accelerometers and digital compasses, which allow for determining the location of a unit using Inertial Navigation System technology. An Inertial Navigation System determines the location of an object relative to an initial location without an external reference frame by using the velocity (measured by the accelerometer) and attitude (measured by the gyroscope and compass) of the object [32]. The combination of these sensors with GPS-based locations has opened up the possibility to determine the location of a unit in-between fixes, and periodically update the accumulated location Inert Navigation System error using the coordinates of successful GPS fixes [4,67–69]. Additionally, it allows for detailed behavioral data to be gathered. The technique has tremendous potential for further development.

## Conclusion

As the golden age of animal tracking science takes off, frequent large-scale evaluations of the techniques used, such as ours, are a necessity. We show that technological advances and product improvements seem to have increased success rates over the years, but that there is still considerable scope for improvement. Scientists, researchers and manufacturers are starting to take advantage of the knowledge generated through field experiences and are working on ways to efficiently deal with the generated data. This also means that we are gaining insights in how to achieve further improvements. With this study, and in all our recommendations, we want to highlight the exciting opportunity for closer collaboration between manufacturers, scientists and wildlife managers to find creative ways to solve any current and future problems encountered. An interesting example is the ICARUS Initiative (International Cooperation for Animal Research Using Space), a global animal observation system using satellite telemetry tags that communicate with ground-based stations through hardware installed on the International Space Station. Eventually, improved design and performance of satellite telemetry units will reduce the impacts of the units on animal welfare, will allow researchers to do better science, will increase the use of the technology across a broad spectrum of biological questions, and will ultimately also lead to better conservation and management decisions.

## Supporting information

S1 Code R-code for boosted beta regression (Fix acquisition rate). (R)

S2 Code R-code for boosted beta regression (Overall fix success rate). (R)

S1 Data Global dataset for boosted beta regressions. (CSV)

S2 Data Description of data fields in S1 Data. (CSV)

S1 Fig Satellite telemetry articles published. (PDF)

S2 Fig Distribution of response variables and covariates. (PDF)

S3 Fig Covariate partial effects on the variability of the fix acquisition rate. (PDF)

S4 Fig Covariate partial effects on the variability of the Overall fix success rate. (PDF)

S1 Questionnaire Standardized data collection questionnaire. (PDF)

S1 Table Satellite telemetry evaluations. (PDF)

S2 Table Tagged individuals per species. (PDF)

S1 Text Trends in observed data. (PDF)

S2 Text Unit purchase and operation costs. (PDF)
