# Supplementary material for: Right on track? Performance of satellite telemetry in terrestrial wildlife research
Source: PLoS One. 2019 May 9;14(5):e0216223. doi: 10.1371/journal.pone.0216223 (PMC6508664; doi:10.1371/journal.pone.0216223)

S1 Fig. Satellite telemetry articles published. Number of publications over the last 17 years found on ISI Web of Knowledge using the search terms ‘satellite wildlife telemetry’, ‘satellite wildlife tracking, ‘GPS wildlife telemetry’, ‘GPS wildlife tracking’, ‘satellite animal telemetry’, ‘satellite animal tracking’, ‘GPS animal telemetry’, and ‘GPS animal tracking’.

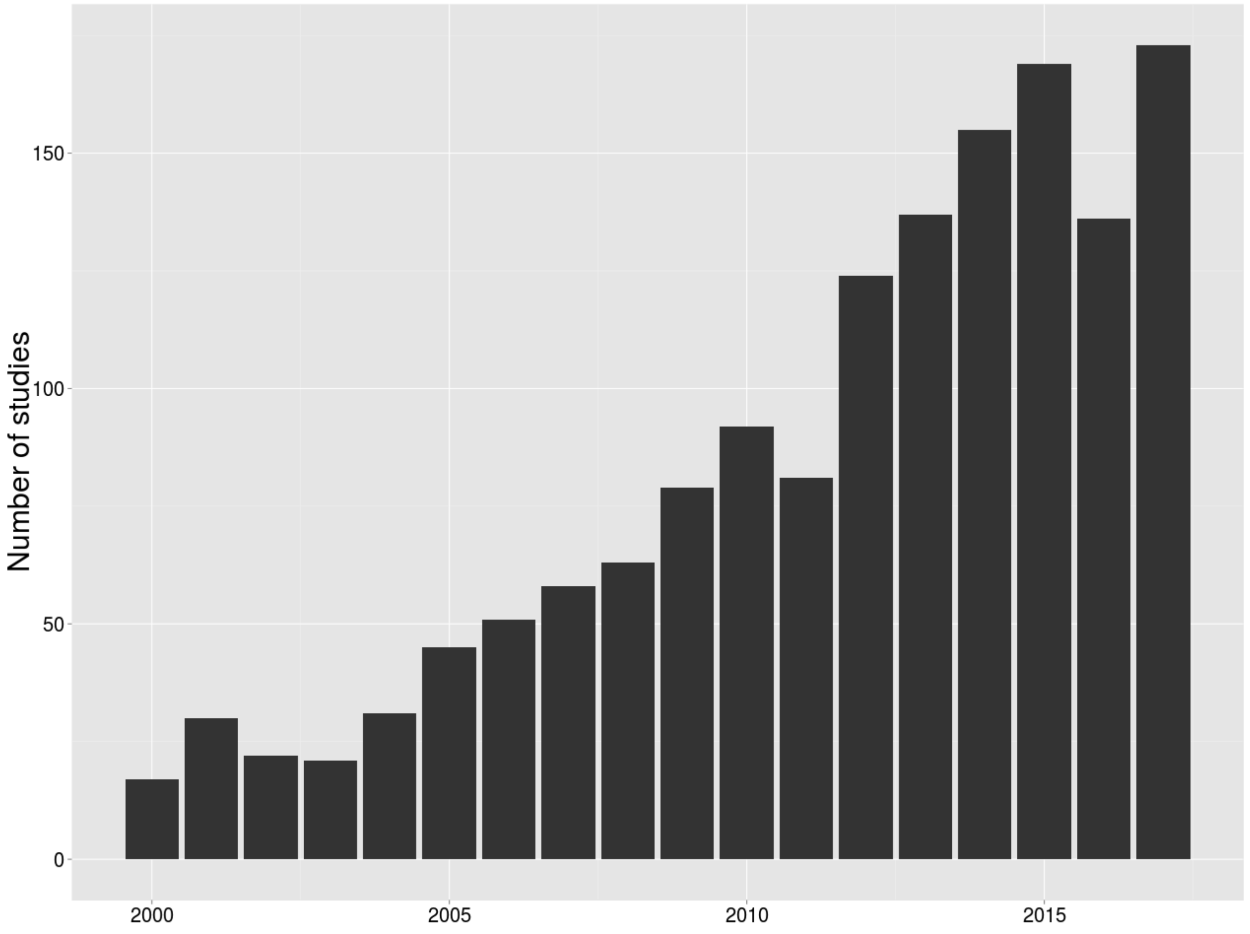

Supplement: S1 Fig — (PDF) [file pone.0216223.s005.pdf]
