# Supplementary material for: Right on track? Performance of satellite telemetry in terrestrial wildlife research
Source: PLoS One. 2019 May 9;14(5):e0216223. doi: 10.1371/journal.pone.0216223 (PMC6508664; doi:10.1371/journal.pone.0216223)
Supplement: S2 Fig — (PDF) [file pone.0216223.s006.pdf]

S2 Fig. Distribution of response variables and covariates. We refer to the Materials and Methods section of the main text for more information regarding the response variables and the covariates (see also Table 1).

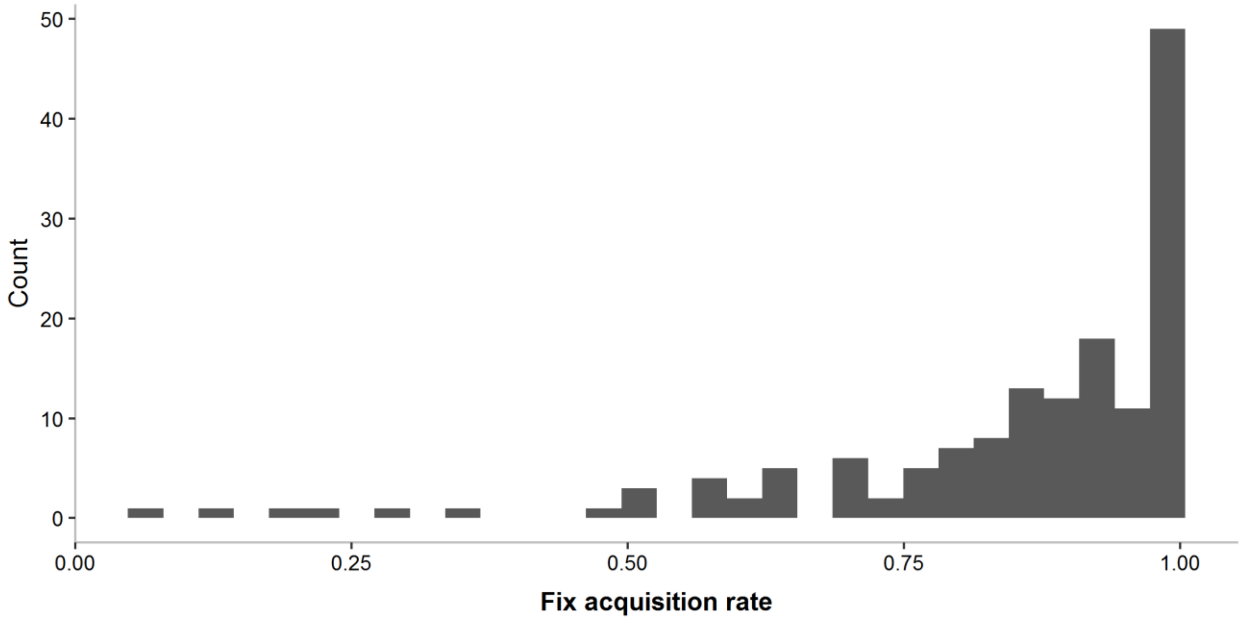

Fig S2.1. Distribution of the Fix acquisition rate

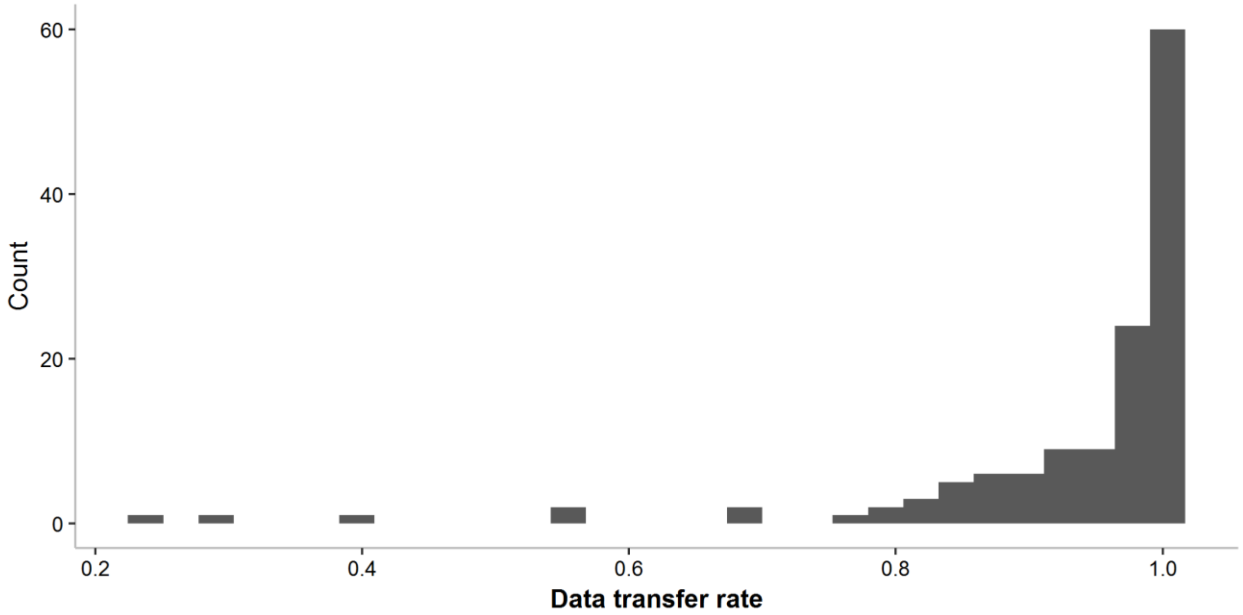

Fig S2.2. Distribution of the Data transfer rate

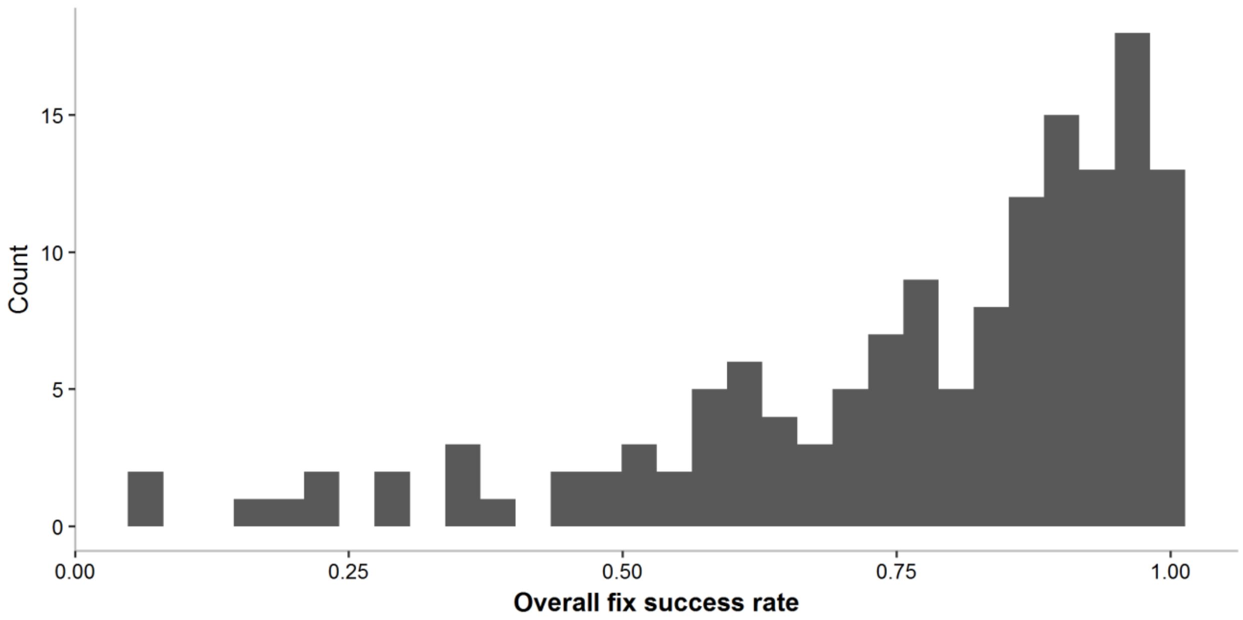

Fig S2.3. Distribution of the Overall fix success rate

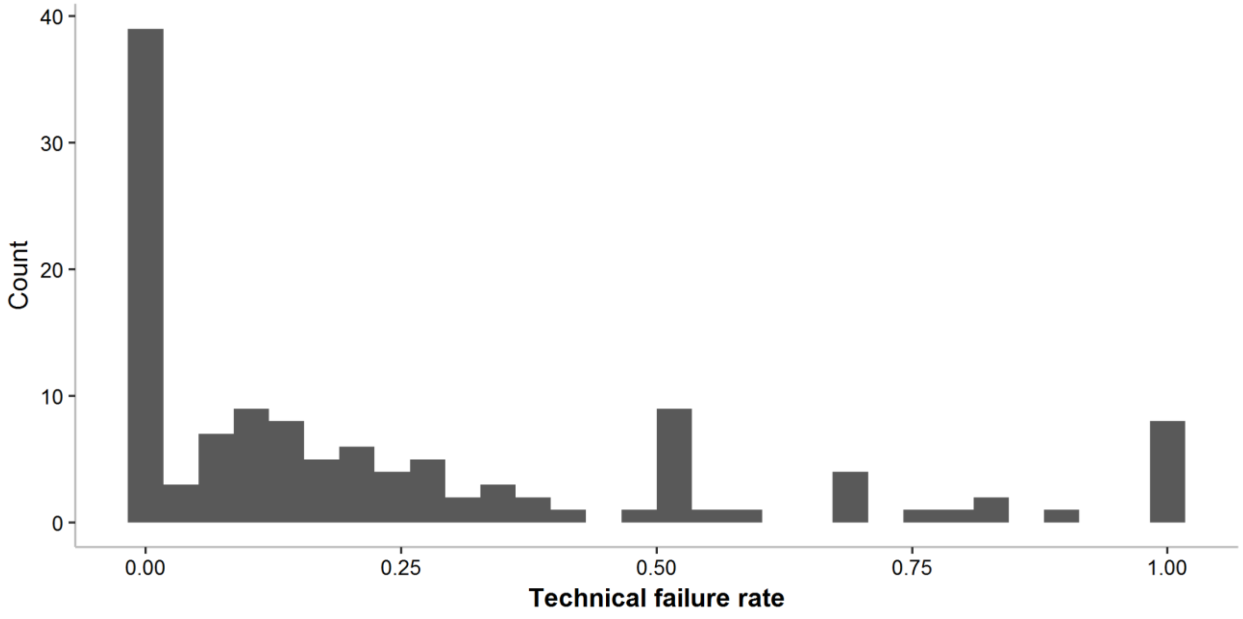

Fig S2.4. Distribution of the Technical failure rate

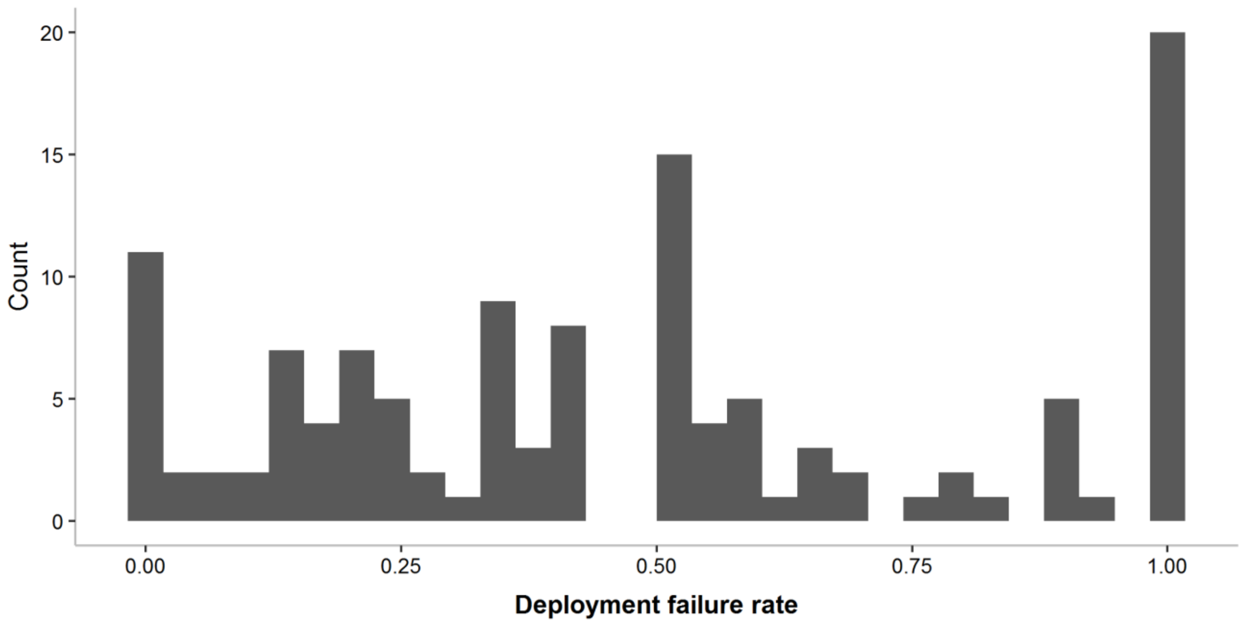

Fig S2.5. Distribution of the Deployment failure rate

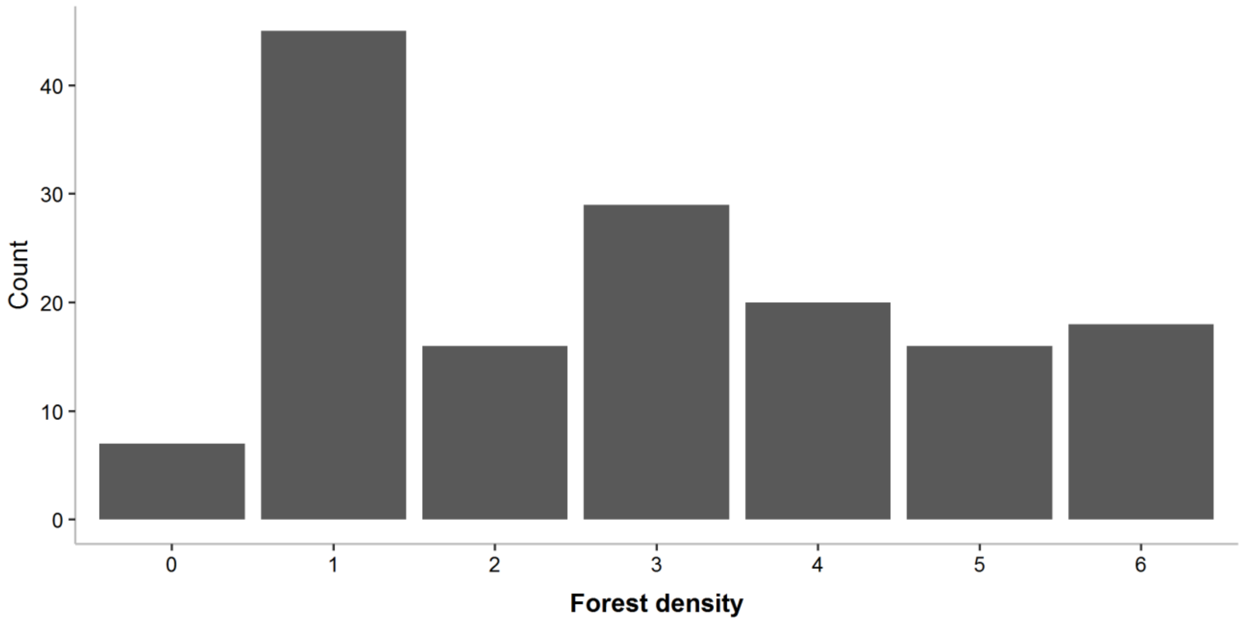

Fig S2.6. Distribution of Forest density. Levels: 0 = No forest cover; 1 = Open understory & sparse canopy cover; 2 = Dense understory & sparse canopy cover; 3 = Open understory & intermediate canopy cover; 4 = Dense understory & inter-mediate canopy cover, 5 = Open understory & closed canopy; 6 = Dense understory & closed canopy.

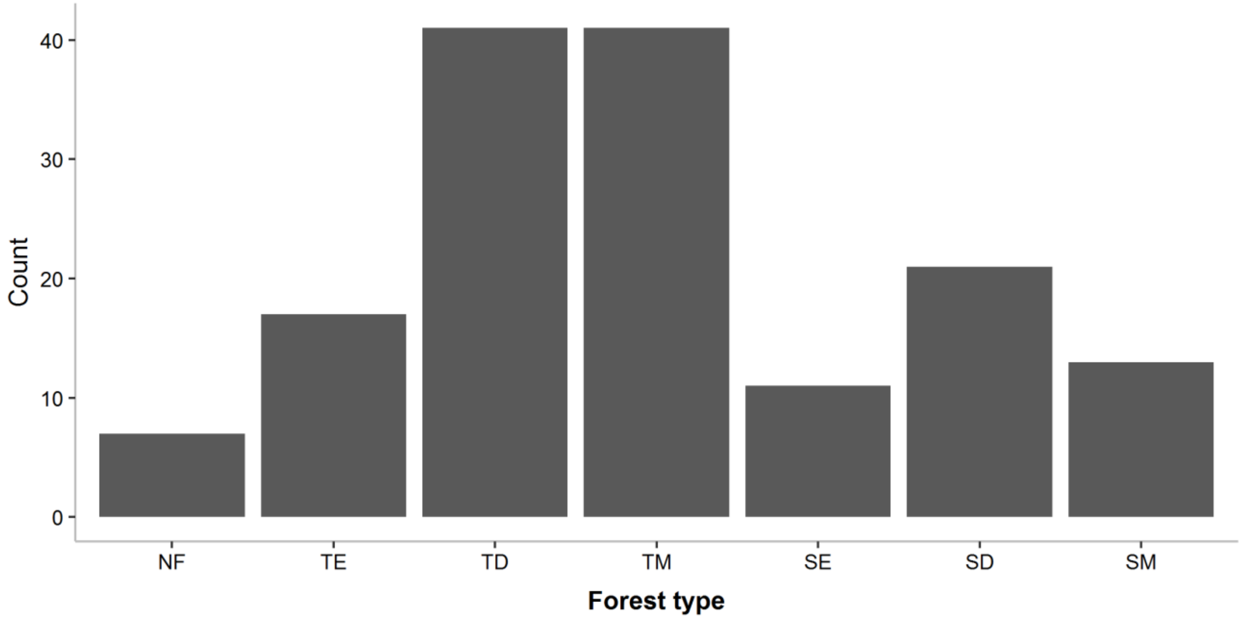

Fig S2.7. Distribution of Forest type. Levels: No forest cover; Temperate evergreen; Temperate deciduous; Temperate mixed; (Sub)Tropical evergreen; (Sub)Tropical deciduous; (Sub)Tropical mixed.

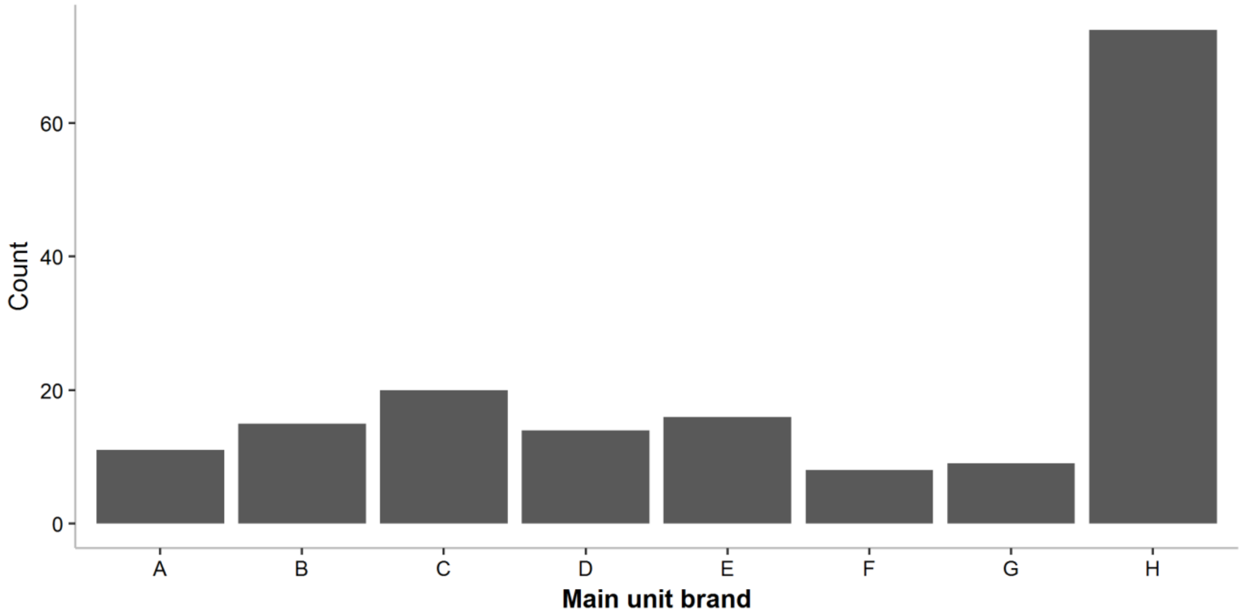

Fig S2.8. Distribution of Main unit brand.

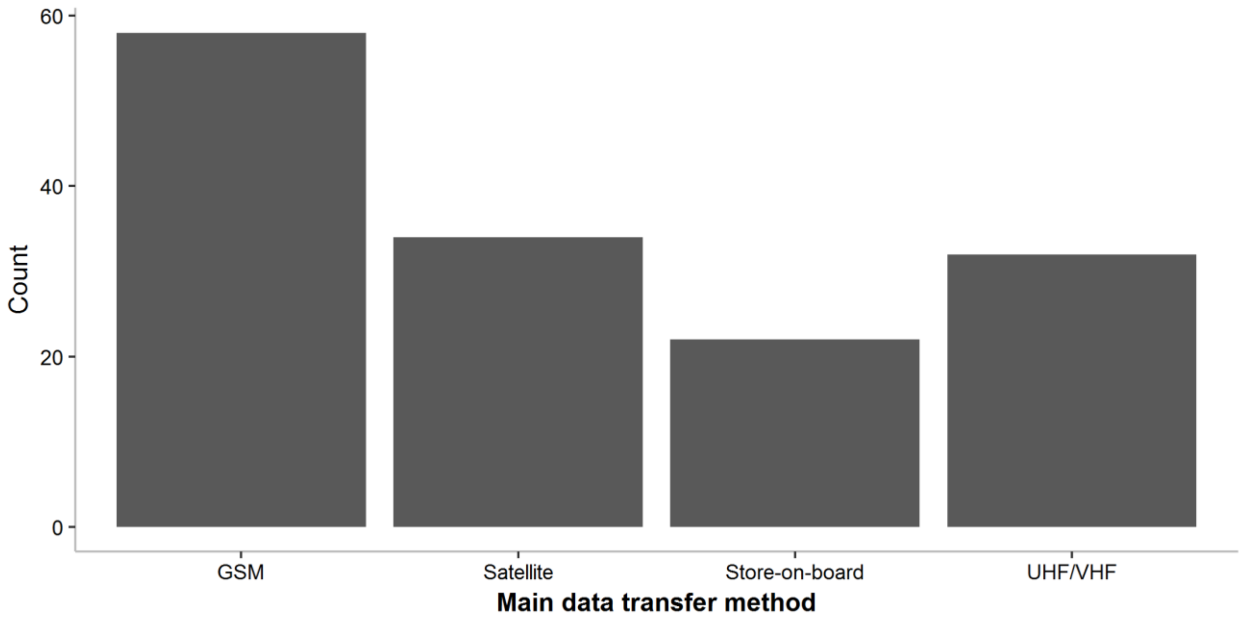

Fig S2.9. Distribution of Data transfer method

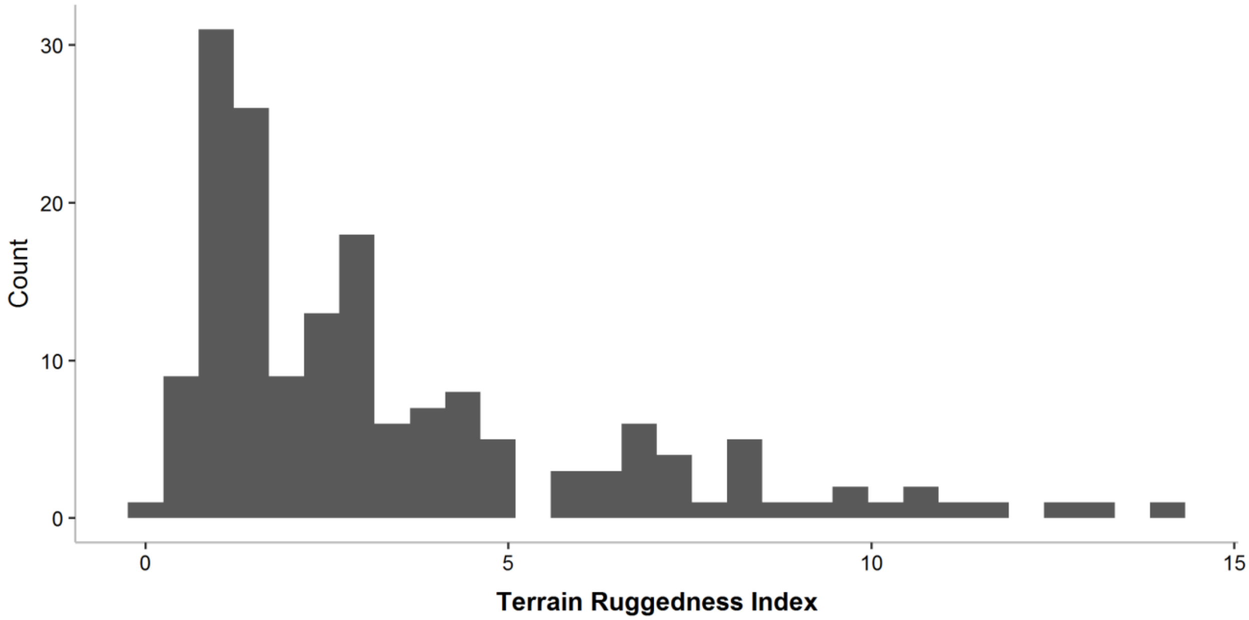

Fig S2.10. Distribution of Terrain Ruggedness Index

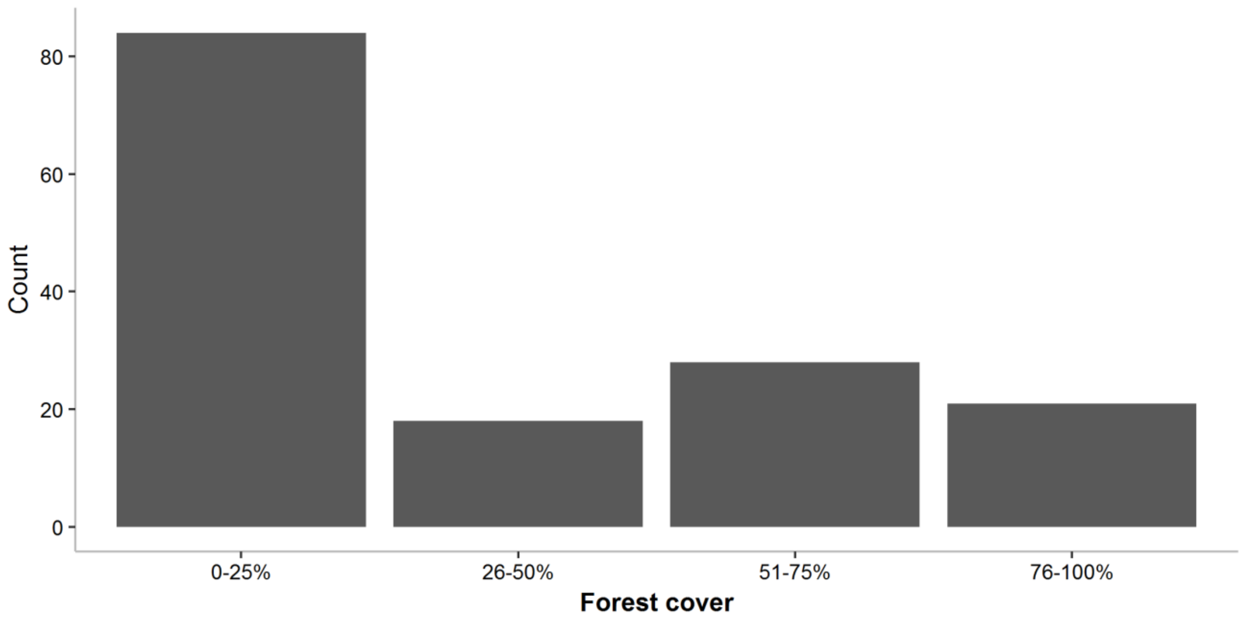

Fig S2.11. Distribution of Forest cover (qualitative).

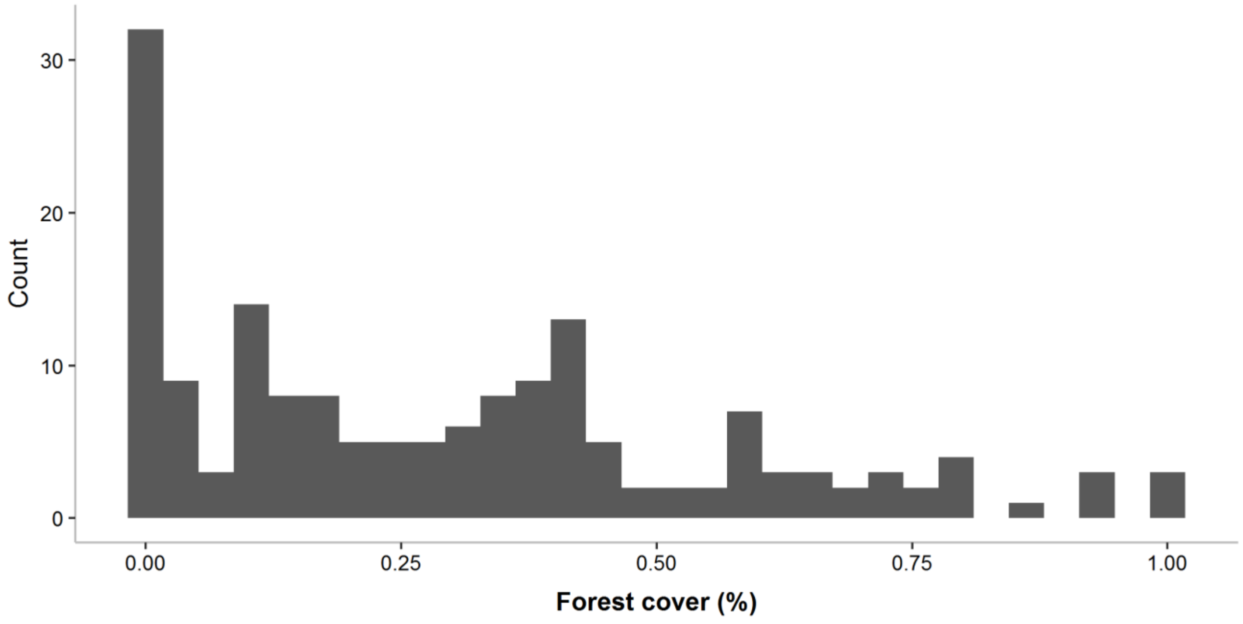

Fig S2.12. Distribution of Forest cover (quantitative)

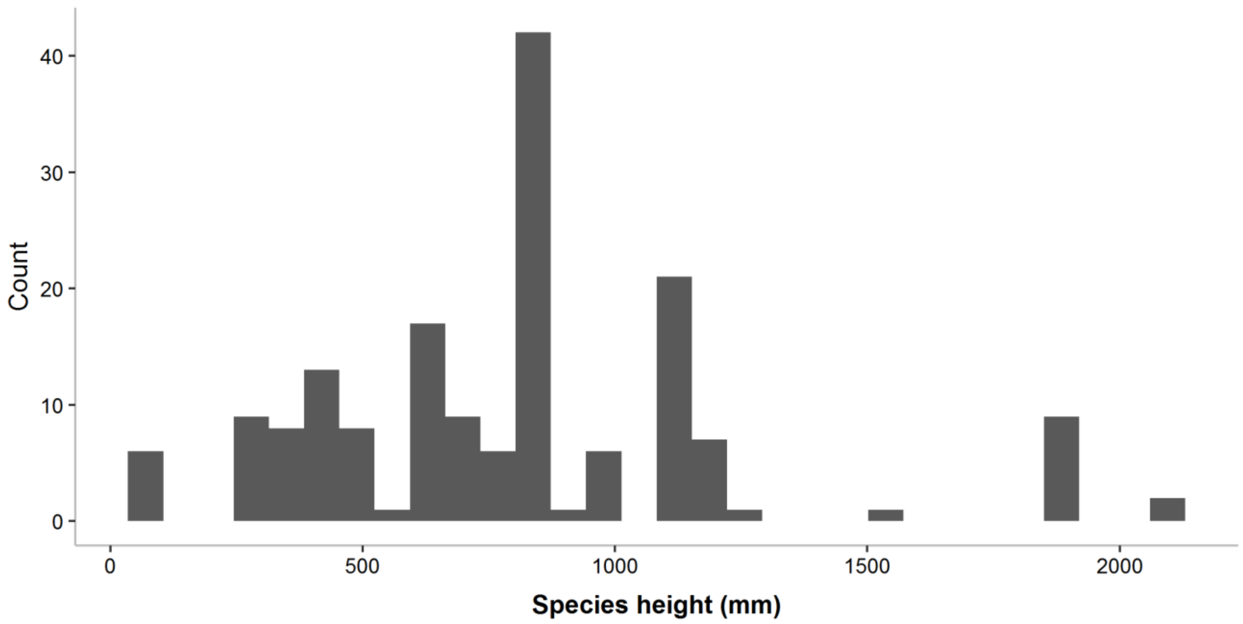

Fig S2.13. Distribution of Species height.

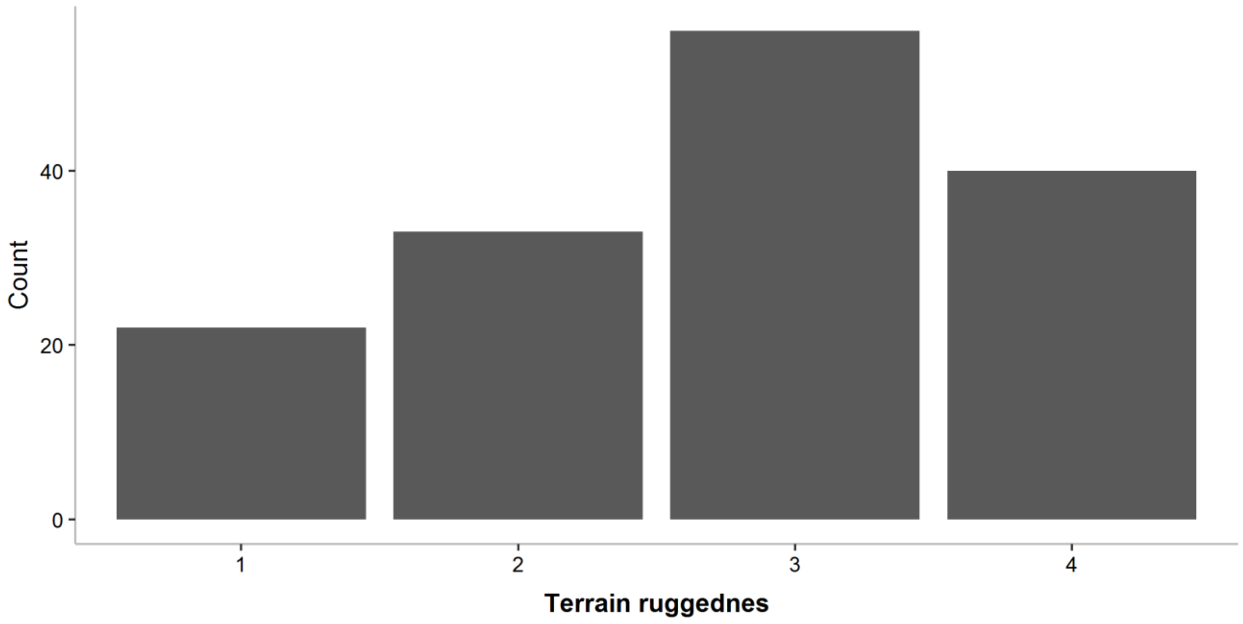

Fig S2.14. Distribution of Terrain ruggedness. Levels: Steep slopes and narrow valleys, flat areas and gentle slopes are rare (< 20%); Steep slopes, interspersed with flat areas and/or gentle slopes; Mostly flat area and/or gentle slopes, with occasional steep slopes (< 20%); Mostly flat area or gentle slopes (< 5% steep slopes).

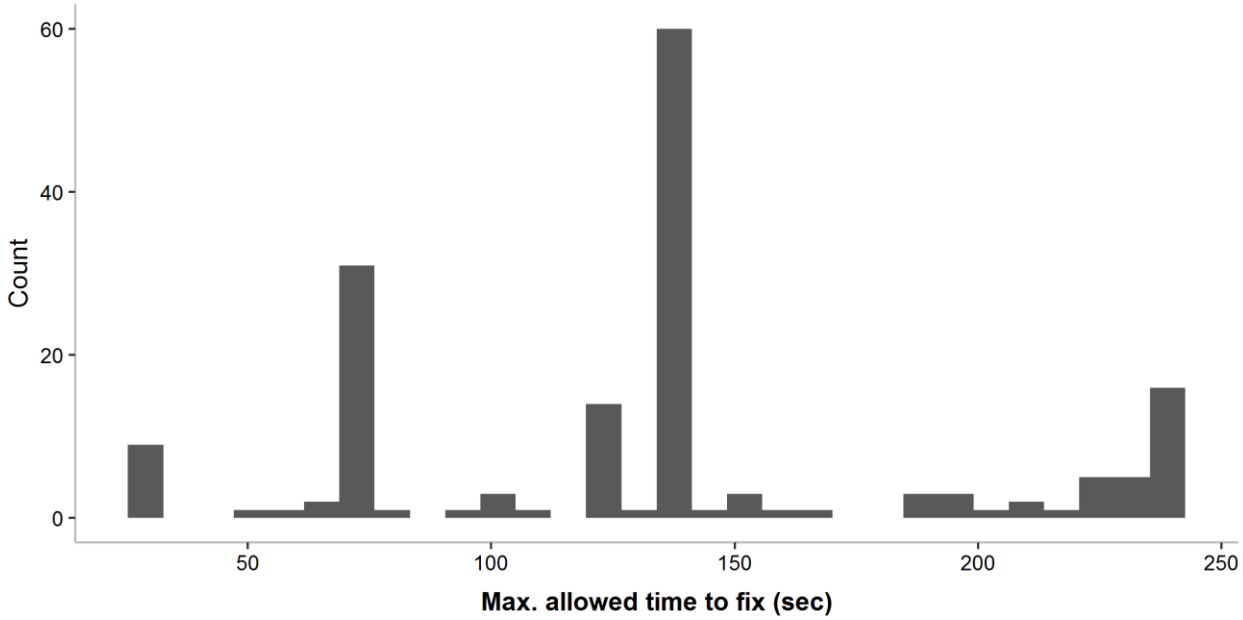

Fig S2.15. Distribution of Maximum allowed time to fix (sec).

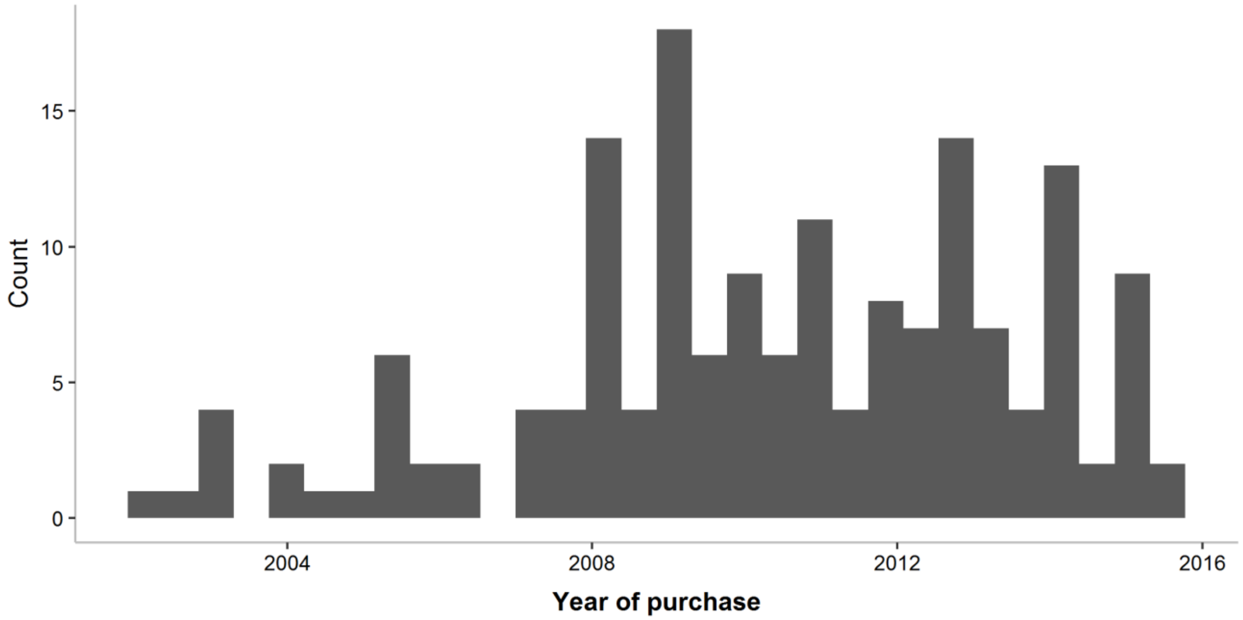

Fig S2.16. Distribution of Year of purchase.
