# Supplementary material for: Right on track? Performance of satellite telemetry in terrestrial wildlife research
Source: PLoS One. 2019 May 9;14(5):e0216223. doi: 10.1371/journal.pone.0216223 (PMC6508664; doi:10.1371/journal.pone.0216223)

S3 Fig. Covariate partial effects on the variability of the Fix acquisition rate. Mean-centered partial effects of the most important variables predicting the variability (φ) of the fix acquisition rate of satellite telemetry units (empirical confidence intervals in grey). Graphs are presented left-to-right in order of importance. Partial effects display the effect of the variable while accounting for all other variables in the model.

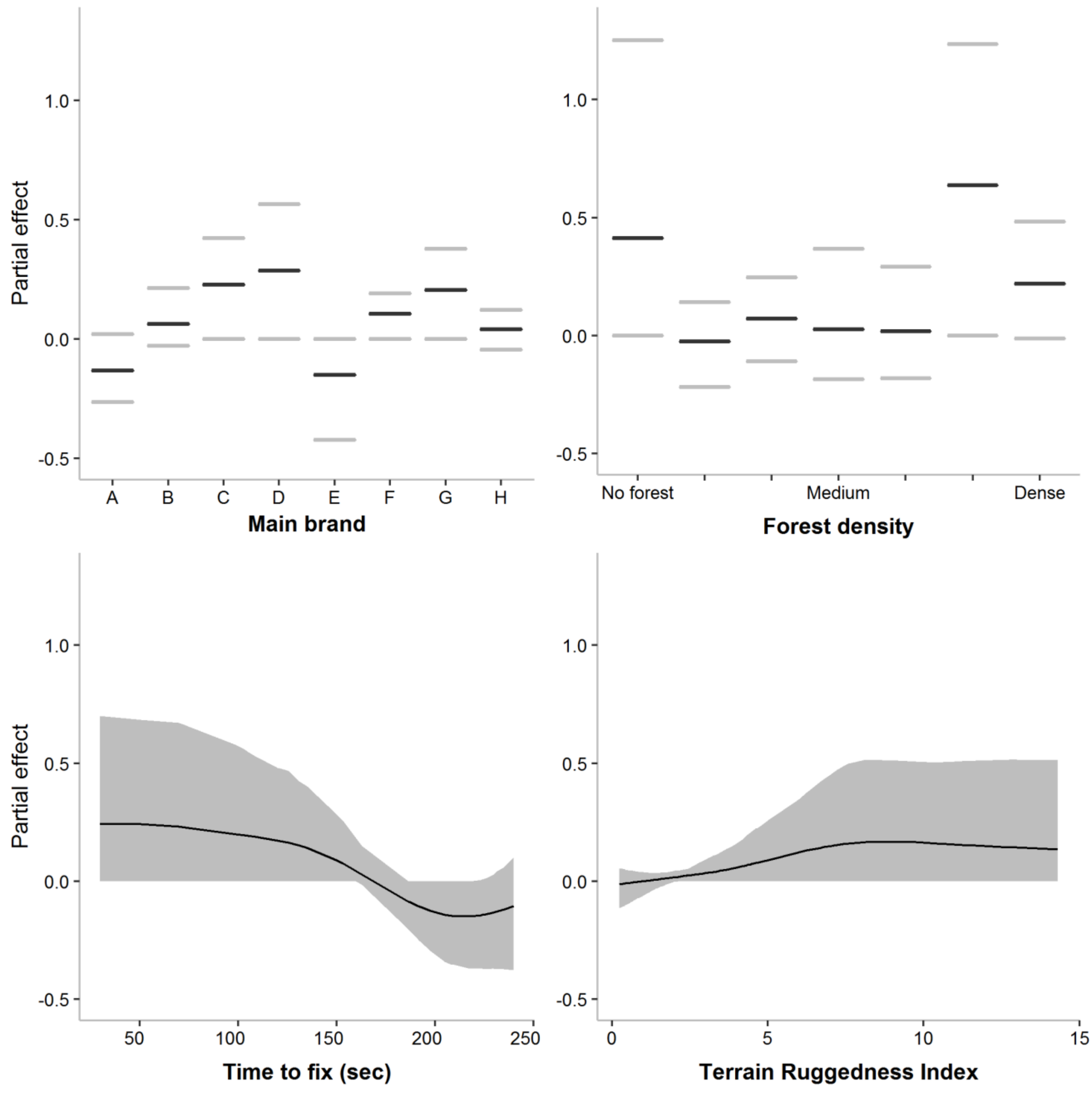

Supplement: S3 Fig — (PDF) [file pone.0216223.s007.pdf]
