# Supplementary material for: Right on track? Performance of satellite telemetry in terrestrial wildlife research
Source: PLoS One. 2019 May 9;14(5):e0216223. doi: 10.1371/journal.pone.0216223 (PMC6508664; doi:10.1371/journal.pone.0216223)

S4 Fig. Covariate partial effects on the variability of the Overall fix success rate. Mean-centered partial effects of the most important variables predicting the variability (φ) of the overall fix success rate of satellite telemetry units (empirical confidence intervals in grey). Graphs are presented left-to-right in order of importance. Partial effects display the effect of the variable while accounting for all other variables in the model. Forest types are: No Forest (NF), Temperate Evergreen (TE), Temperate Deciduous (TD), Temperate Mixed (TM), (Sub)Tropical Evergreen (SE), (Sub)Tropical Deciduous (SD), and (Sub)Tropical Mixed (SM).

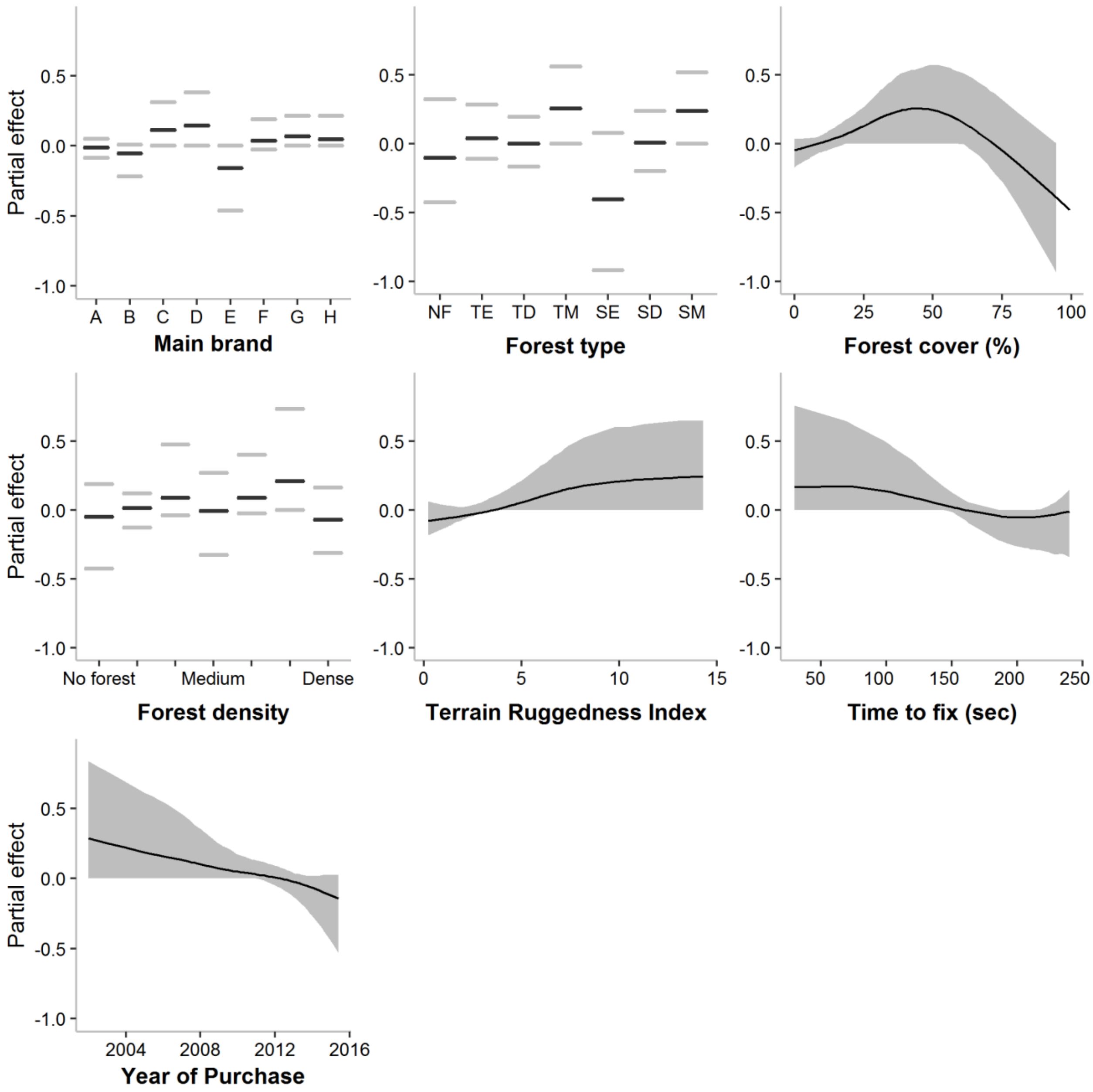

Supplement: S4 Fig — (PDF) [file pone.0216223.s008.pdf]
