# Supplementary material for: Right on track? Performance of satellite telemetry in terrestrial wildlife research
Source: PLoS One. 2019 May 9;14(5):e0216223. doi: 10.1371/journal.pone.0216223 (PMC6508664; doi:10.1371/journal.pone.0216223)
Supplement: S1 Questionnaire — (PDF) [file pone.0216223.s009.pdf]

GEORG-AUGUST-UNIVERSITÄT GÖTTINGEN

PRIFYSGOL BANGOR UNIVERSITY

PERFORMANCE OF SATELLITE COLLARS IN WILDLIFE RESEARCH: WHAT DOES THE EVIDENCE SHOW?

A survey by Maarten P.G. Hofman[1,2], Matthew W. Hayward[2], Julia P.G. Jones[2] and Niko Balkenhol[1]
[1] Dept. of Wildlife Sciences, University of Göttingen, Germany
[2] School of Environment, Natural Resources and Geography, Bangor University, Wales, UK

Dear colleague,

Telemetry collars equipped with GPS functionality are a very attractive option in wildlife research, because they allow for tracking of movement and activity on a very fine temporal and spatial scale. However, one limitation of this technology is that GPS fix success rates and positional errors are highly variable, and are affected to unknown extent by collar orientation, canopy cover, terrain ruggedness, etc. These confounding factors often cause bias in the locations obtained, and in some cases might prevent the device from obtaining sufficient data to answer research questions, forcing to change or abandon project objectives. This has led some authors to urge for caution when opting for satellite collars. Unfortunately, most unsuccessful studies do not get published, their analysis not completed and/or their results discarded. The literature therefore is biased towards successful applications of satellite collars, giving the general impression that the technique is useful under most circumstances. With this survey, we want to give the chance to such unsuccessful studies to still contribute to the field of wildlife research. We want to tap into both unsuccessful and successful research projects using satellite collars to see if we can find out whether there is an identifiable set of circumstances under which the technique should be a safe bet to yield useful data and/or under which circumstances it might be advisable to look for alternative methods to answer the questions at hand.

The survey will likely require you to dig up data and/or publications and perform some calculations, but we hope that this will not discourage you from contributing to the assessment of satellite collars as a wildlife research technique. Filling out the survey will take anywhere between 20 minutes and half a day, depending on the size of your dataset and how close you have your data at hand.

The survey will be closed by May 31th, 2016.

If you wish so, you will receive a summary of the results when the study is finished. In this case, please indicate so in the form on the next page.

We thank you for taking this survey, and hope you will bear with us as we guide you through the questions. Throughout the form, questions marked with an asterix (*) need to be filled out in order for your project to be used in the analyses.

Let's start!

First, we would like to get a minimal amount of background data on our respondents. Remember that we will take into account the preference you have indicated in our online form regarding your personal details to be permanently removed at least 5 months after the end of the study.

Please provide your personal information.
(All personal details will be treated strictly confidential and will only be used for this survey. Note that you can choose not to fill out your personal details, in which case all your deployment data will be treated as strictly anonymous throughout the study.)

Last name ______ **First name** ______

Affiliation ______

	Yes	No
**Would you like to be informed about the results when the study is finished?** (If yes, please provide contact details below)	○	◉
**Can we contact you for further information, if necessary?** (If yes, please provide contact details below)	○	◉
**Would you like to remain anonymous in any acknowledgements for this study?**	◉	○

Contact details
(Note that you can choose to leave the fields blank. In this case, you will not be informed about the results or contacted for further information about the data you provide.)

Email ______ **Phone** ______

Thank you! This got us started.
Please save this document for later submission. We will now go on to input the deployment data.

We have assumed most studies will have the requested data stored on a project basis. Hence, we have chosen the project as the level of data entry. By clicking the "Add project data" button below, you will be prompted to download a PDF form from the server at the University of Göttingen. Bear in mind that most browsers will not allow you to fill out the form inside the browser. You will need to save it to your local computer and open it with the latest version of your PDF viewer. Our Linux server is clean, safe and well-maintained; you can trust the file for download. The form will allow you to enter all collar deployment information pertaining to one project. If you have conducted more than one project, you will have the opportunity to start adding the data for any additional project(s) at the end of the downloaded form.

Please save this form and press the "Add project data" button to start entering your deployment data.

Add project data

For more information about this survey:
Maarten Hofman, Department of Wildlife Sciences, University of Göttingen
Büsgenweg 3, 37077 Göttingen, Germany
+49 (0)551 39 33583 - maarten.hofman@forst.uni-goettingen.de

GEORG-AUGUST-UNIVERSITÄT
GÖTTINGEN

PRIFYSGOL
BANGOR
UNIVERSITY

PERFORMANCE OF SATELLITE COLLARS IN WILDLIFE RESEARCH: WHAT DOES THE EVIDENCE SHOW?

A survey by Maarten P.G. Hofman[1,2], Matthew W. Hayward[2], Julia P.G. Jones[2] and Niko Balkenhol[1]
[1] Dept. of Wildlife Sciences, University of Göttingen, Germany
[2] School of Environment, Natural Resources and Geography, Bangor University, Wales, UK

PROJECT INFORMATION FORM

Throughout the form, questions marked with an asterix (*) need to be filled out in order for your project to be used in the analyses.

I. PROJECT AREA

To start off, we would like the location and extent of the project area to be filled out in the table below. Please, bear in mind the following:

- If your project involves collars deployed in disjunct areas and/or under clearly distinct environmental circumstances, please consider filling out separate forms for each of these areas.
- If your project area(s) cross(es) an international border, you can list all countries involved.
- The extent is indicated by the geographic (non-projected) coordinates of the lower left and upper right corner of the rectangle that best describes your project area. Coordinates need to be in the WGS84 coordinate system and in decimal degrees format (see Figure 1 and the first row of the Project area table).

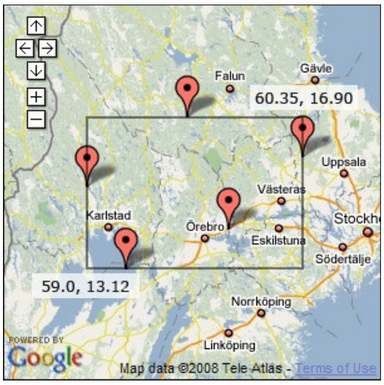

Figure 1: Example map illustrating the measurement of the project area extent

Assistance
To convert your project area extent from your local coordinate system to WGS84 (GPS), you can use this online tool.
To find out the coordinates of a point in Google Maps, right click the point and choose "What's here?" from the pop-up context menu.

1. Area name*	Country*	Latitude 1*	Longitude 1*	Latitude 2*	Longitude 2*
Example area Örebro**	Sweden	59.0000	13.1200	60.3500	16.9000

** see Figure 1

Now we would like to get a general impression of the environmental situation in your project area.

2. Forest cover in the landscape*	
3. Dominant forest type*	
4. Typical forest density in the area*	
5. Terrain ruggedness*	

Importantly, we also need to know the time span over which the project was conducted.

6. Project period from* dd/mm/yyyy **until*** dd/mm/yyyy

II. Species

Please enter the number of individuals of all species for which deployment data is provided. If possible, group the counts per sex and age combination. Please contact us if more species have been collared than fit in the provided fields.

7. Colloquial name	Scientific name*	Sex	Age	Number*
				0
				0
				0
				0
				0
				0
				0
				0
Total				0

III. Collars

We are now getting closer to the core information needed, and dive a little deeper into the characteristics of the collars. In the table below, please provide the number of collars deployed per brand (and type, if possible) and year of purchase. Again, please contact us if more collars have been deployed than fit in the provided fields.

8. Brand*	Type	Year of purchase*	Number*
			0
			0
			0
			0
			0
			0
			0
			0
			0
Total			0

Before we go on with some further information on the collars, please have a look at the following diagram to clarify the terminology used. What is the fix attempt time and when is a fix successful?

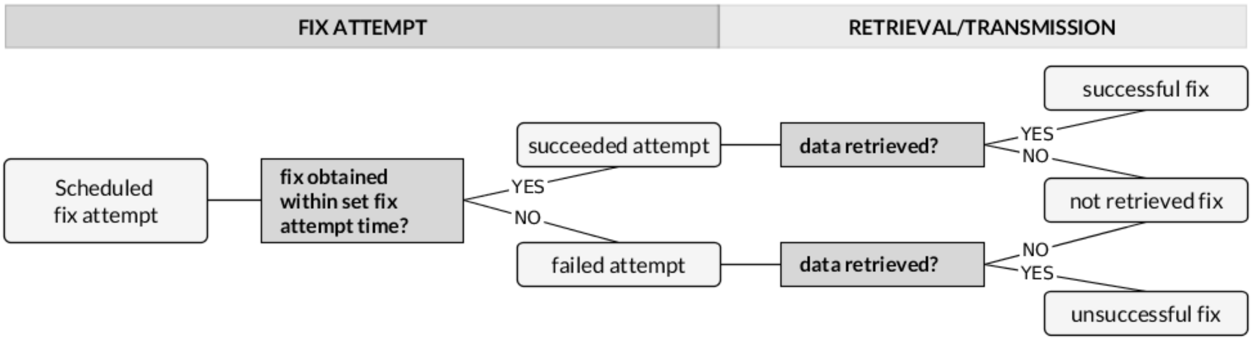

Figure 2: From fix attempt to usable geographic information. When is a fix successful?

The **fix attempt time** (the first step in Figure 2) is defined as the time span over which a collar is allowed to search for sufficient satellites to obtain a fix before the attempt is classified as failed. A fix attempt is considered **successful** if it (i) succeeded in obtaining the collar's geographical position within the set fix attempt time and (ii) was successfully transmitted/retrieved from the collar. A fix attempt is labelled **unsuccessful** if it failed to obtain a position, and it is labelled '**not retrieved**' when information on its status (succeeded/failed) could not be obtained from the collar (Figure 2). There are three general ways of **obtaining data** from a collar (the second step in Figure 2 - retrieval/transmission). First, data can be obtained directly from the collar upon its recovery from the field. Second, data can be retrieved remotely using regular VHF/UHF or GSM systems. And third, data is transmitted through satellite-based systems, e.g. Argos, Globalstar or Iridium, and then forwarded to the end user, usually via email.

9. For how many collars was the programmed maximum fix attempt time of the following length?

Fix attempt time	Number
**≤ 60sec**	0
**61-90sec**	0
**91-180sec**	0
**> 180sec**	0
**Unknown**	0

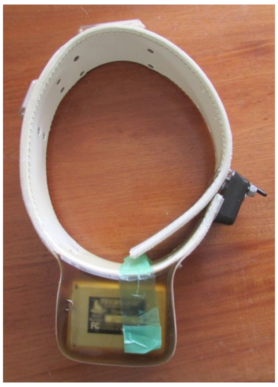

10. How many collars** used the following methods to retrieve the GPS fixes (see text above)?

Data retrieval method		Number
**Upon collar retrieval**		0
**VHF/UHF**		0
**GSM**		0
**Argos**		0
**Globalstar**		0
**Iridium**		0
**Unknown**		0
**Other**	Please specify	0

**Please include all deployed collars, regardless of whether they actually yielded fixes or not.

IV. Deployment

Good. So far we are aware of the number and type of collars that were deployed at the project site for any duration within the project period. Now, we need to know some more deployment details. How often was data transmitted? How many fix attempts were obtained? How did the deployment end? Etc.

11. For all collars using satellite data transmission, how many sent the obtained positions to the satellite at the following frequencies?

no collars with satellite data transmission were used ☐

**more than 1 transmission attempt per day**		0
**1 transmission attempt every 2-3 days**		0
**1 transmission attempt every 4-7 days**		0
**1 transmission attempt every 2-4 weeks**		0
**less than 1 transmission attempt per month**		0
**Unknown**		0
**Other**	Please specify	0

12. For how many collars did the deployment period end as follows? (Please include all deployed collars)

**It hasn't ended yet**		0
**As planned (drop-off, battery dead, collar retrieved, project end)**		0
**Unexpected battery failure**		0
**Electronic failure (software/sensor/electronics)**		0
**Mechanical failure (e.g. collar damaged)**		0
**Unknown (e.g. collar lost)**		0
**Other**	Please specify	0

13. From all collars with remote data retrieval (VHF/UHF, GSM or satellite), how many yielded additional successful fixes after they were retrieved from the field? 0

We have now arrived to the key number of the questionnaire: the **fix success rate**. We calculate the fix success rate as the proportion of all scheduled fix attempts that was successful, as defined in Figure 2. Therefore, we need the number of expected (scheduled) fixes, the number of fixes that could not be retrieved, and the number of successful fixes.

14. Over all deployed collars, what was the total number of *expected fixes* over the project period? That is, when all scheduled fix attempts over the project period would have been successful, how many fixes would there have been?* 0

15. Over all deployed collars, how many scheduled fix attempts were *not retrieved* (e.g . because of poor satellite connection, failing GSM network, failing VHF or just data loss)? That is, the status (failed/succeeded) of these fixes is unknown.* 0

16. Over all deployed collars, how many *successful fixes* were eventually obtained over the project period, regardless of positional error (e.g. DOP) or number of satellites involved?* 0

V. PROJECT OPERATION AND EVALUATION

We are now nearing the end of the questionnaire, and would like to know some more details about the operation and evaluation of the project. For example, what are the average costs involved in projects using satellite collar techniques. We are only interested in broad cost classes to investigate the profitability of the approach under different environmental circumstances and varying fix success rates.

17. What was the average purchase price range per collar?
(including drop-off mechanism, shipping and/or import costs, excluding accessories and software)

18. What were the average additional *non-recurring* costs per collar?
(e.g. additional hardware, software, etc.)

19. What were the average *recurring* running costs *per collar per month*?
(e.g. satellite service, GSM network, battery replacement)

Knowing you have invested these resources into your project hoping for helpful and insightful results, we want to gauge to what extent you were satisfied with the amount and quality of the data you obtained using the satellite collar technique.

20. How well do the following two statements reflect your impressions about the collars in your project?

	Very poor	Poor	Neutral	Good	Very good	I don't know
The amount of data obtained (i.e. fix success rate and transmission success) met my expectation.	○	○	○	○	○	○
The data quality (position error) met my expectation.	○	○	○	○	○	○

And finally, we are interested whether the results of this project were useful for ecological or conservation research? Was the data useful for furthering your career? Was it used in publications?

21. How well do the following statements reflect your impressions on the usefulness of the project results?

	Very poor	Poor	Neutral	Good	Very good	I don't know
The project results were useful for applied conservation.	○	○	○	○	○	○
The project results were useful for the field of ecological or conservation research I am active in.	○	○	○	○	○	○
The project results were useful for furthering my career.	○	○	○	○	○	○

22. How many publication(s) report on the data from these collars?

Peer-reviewed journal publications 0

Other publications 0

23. Please provide citations for any peer-reviewed journal publications mentioned in Question 22.

24. If none, why did the project not result in any publications?

25. Have you uploaded any of your data to Movebank?

◯ Yes ◯ No

Movebank is a free, online database of animal tracking data hosted by the Max Planck Institute for Ornithology, helping researchers to manage, share, protect, analyse, and archive their data, including unpublished datasets.

26. If you have not done so, what was the most important reason?

VI. REMARKS AND FURTHER INFORMATION

27. If you have any further remarks about the project details, specific collar details, etc. please provide them here.

Please verify that you have answered all 27 questions, or at least those marked with an asterix (*).
If so, you have provided all necessary deployment data for your study to be included in our analysis, and you will find out how you can submit this form on the next page.

THANK YOU!

If you have conducted any additional projects and you would like to add their deployment data as well, you can use the “Add another project” button below to start entering the data for these project(s) using a new form.

If you have no further projects to add, send us the filled out form(s) following these instructions:

- save this project information form under a unique name (e.g. the name of the project area),
- combine this project information form (and those for any additional projects) with your previously filled out personal information form in a compressed folder (e.g. .zip), and
- send the compressed folder to maarten.hofman@forst.uni-goettingen.de with in the subject line: “Satellite collars in wildlife research”

To add another project, please save this form as mentioned above and press the “Add another project” button to start entering the deployment data of your next project.

Add another project

For more information about this survey:
Maarten Hofman,
Department of Wildlife Sciences,
University of Göttingen
Büsgenweg 3, 37077 Göttingen,
Germany
+49 (0)551 39 33583
maarten.hofman@forst.uni-goettingen.de

PERFORMANCE OF SATELLITE COLLARS IN WILDLIFE RESEARCH: WHAT DOES THE EVIDENCE SHOW?

A survey by Maarten P.G. Hofman[1,2], Matthew W. Hayward[2], Julia P.G. Jones[2] and Niko Balkenhol[1]
[1] Dept. of Wildlife Sciences, University of Göttingen, Germany - [2] School of Environment, Natural Resources and Geography, Bangor University, Wales, UK

ADDITIONAL GUIDELINES FOR THE PROJECT INFORMATION FORM

Q1: Ideally, the coordinates should be the bounding box of the observed locations of all collared animals in the project, or the bounding box of their compiled home ranges.

Q2-5: Think about the general area within the bounding box that was actually used by the individuals.

Q6: If some of the collars are still deployed and providing data, the end date of the project should be set to the date up until which you have calculated the expected fixes (see Q14).

Q7: Include all animals that were collared at least once during the project.

Q8: Include all collars that were deployed at least once during the project, regardless of whether they were refurbished. Include the year of original purchase.

Q9: We are interested in the maximum time that the collar was programmed to look for a GPS fix during a fix attempt. Please include all deployments (collar-animal combinations) that occurred during the project. That is, the sum of all numbers in this question should equal the number of deployments during the project.

Q10: We are interested in the mode of data transfer that was originally intended to be used. If the collar is equipped to transfer data using the GSM network, this would be the intended method of data transfer, even if you downloaded most fixes directly from the collar after retrieving it from the field. (All collars from which you downloaded additional fixes after retrieval should be listed in Q13). Please include all deployments (collar-animal combinations) that occurred during the project.

Q11: Please include all deployments (collar-animal combinations) that occurred during the project.

Q12: Please include all deployments (collar-animal combinations) that occurred during the project. Animal mortalities can be included in the “Other” section, but indicate the number of mortalities in the specification.

Q13: What we are after here, is the number of retrieved collars from which you downloaded fixes (e.g. using cable connection to a laptop) that you weren't able to download remotely using VHF/UHF, GSM or a satellite network (Argos, Globalstar, Iridium, …) while the collar was deployed.

Q14: In general, 'Expected' in our questionnaire means: how many fixes could all the collars deployed during the project period have made during the time they were active and functioning according to schedule? That is, every fix attempt scheduled on the collars from the start of deployment till the end of deployment or until failure detection is expected to yield a successful fix. As soon as the collars malfunctioned, we are not expecting to get any fixes from them any more.
For any collars that are still deployed, you can choose a date in the recent past up until which you will calculate the expected fixes for all collars. This will also be the project end date (Q6) for the purposes of our study.

Q15-16:

There are in fact three possible outcomes of a fix attempt (defined in Fig. 2 in the questionnaire):

- **Successful** = GPS position obtained during fix attempt by the collar, and this information was retrieved from the collar (Q16)
- **Unsuccessful** = GPS position NOT obtained during fix attempt by the collar, and this information was retrieved from the collar (calculated from Q14 to Q16)
- **Not retrieved** = Information on the scheduled fix attempt could not be retrieved from the collar. It is unknown whether the collar managed to obtain a GPS position. (Q15)

such that:

Expected fixes (Q14) = Successful fixes (Q16) + Unsuccessful fixes + Not retrieved fixes (Q15)

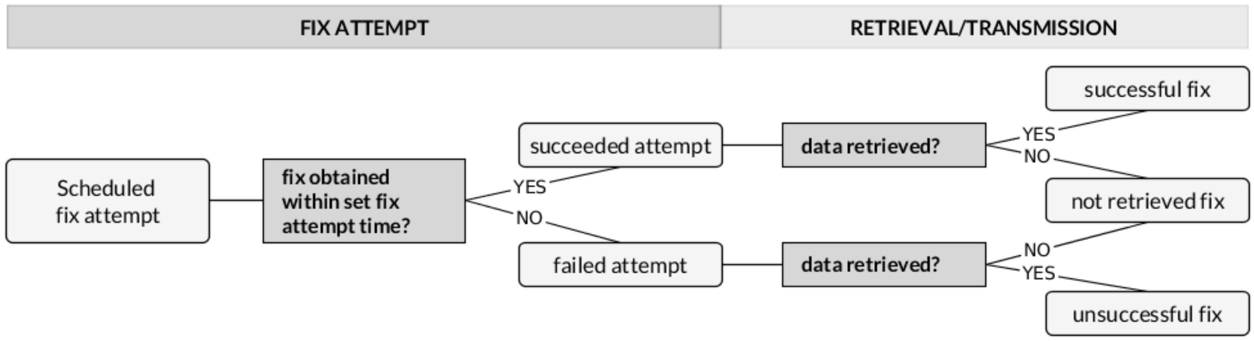

Figure 1: From fix attempt to usable geographic information. When is a fix successful?

An example:

When I get data from my Iridium collars, the files include information on every scheduled fix attempt that the Iridium network managed to download from the collars, regardless of whether it succeeded or not.

That is, the file includes information such as:
- start time of fix attempt
- end time of fix attempt (time to fix) - this will say '>180sec' if the fix attempt failed
- coordinates - these will be blank for a failed fix attempt
- DOP/coordinate precision - again blank for failed attempts
- No. of satellites
- etc.

The fix attempts with coordinates are the **successful** fixes.
From the fix attempts with blank coordinates, I can tell that a fix was **unsuccessful**.
The fix attempts that were scheduled, but are missing from the Iridium files, could not be downloaded from the collar by the Iridium network, and are therefore considered **not retrieved**.

Some collar manufacturers only provide the user with the fix attempts that were successful, while not specifying the fate of the other scheduled fix attempts. In that case you would not be able to distinguish between unsuccessful and not retrieved fixes, and you can leave Q15 blank. Please make note of this in the remarks section (Q27).
