# Supplementary material for: Right on track? Performance of satellite telemetry in terrestrial wildlife research
Source: PLoS One. 2019 May 9;14(5):e0216223. doi: 10.1371/journal.pone.0216223 (PMC6508664; doi:10.1371/journal.pone.0216223)
Supplement: S1 Table — (PDF) [file pone.0216223.s010.pdf]

S1 Table. Satellite telemetry evaluations. List of publications evaluating satellite telemetry, indicating whether success rates or failure rates (as defined in this study) are reported (1) or not (0). *For publications reporting only on stationary tests, the number of collars used in the study is listed; for publications reporting on animal-borne collars, the number of deployments is listed (if reported). **Fix quality may include measures of fix dimensionality (2D, 3D) and/or accuracy (dilution of precision, horizontal error, etc.).

Year	# of deployments or collars*	Stationary/animal-borne	Species	Region	Fix quality**	Fix acquisition rate	Data transfer rate	Overall fix success rate	Deployment failure rate	Technical failure rate	Reference
1995	6	ST/ AB	*Alces alces*	Ontario, Canada	1	1	0	0	0	0	(1)
1996	2	ST/ AB	*Alces alces*	northern Sweden	1	1	0	0	1	1	(2)
1996	1	ST/ AB	*Alces alces*	Minnesota, USA	1	1	0	0	0	0	(3)
1997	?	ST/ AB	*Alces alces*	Minnesota, USA	1	1	0	0	0	0	(4)
2000	5	AB	*Odocoilus virginianus*	Mississipi, USA	1	1	0	0	0	0	(5)
2001	10	AB	*Cervus elaphus nelsoni*	New Mexico, USA	0	1	1	1	1	1	(6)
2001	2	ST/ AB	*Loxodonta africana*	Congo	1	1	1	1	1	1	(7)
2002	26	AB	*Rangifer tarandus caribou*	British Columbia, Canada	1	1	1	1	1	1	(8)
2002	36	ST	---	British Columbia, Canada	1	1	0	0	0	0	(9)
2003	2	ST	---	USA	1	1	0	0	0	0	(10)
2004	81	AB	*Ursus arctos*	western and northern Canada	1	1	1	1	1	1	(11)
2004	1	AB	*Macaca fuscata*	Chiba Prefecture, Japan	1	1	0	0	1	1	(12)
2004	7	ST/ AB	*Cervus elaphus*	Cévennes, France	1	1	0	0	0	0	(13)
2005	?	ST	---	southwestern Arizona	1	1	0	0	0	0	(14)

Year	# of deployments or collars*	Stationary/animal-borne	Species	Region	Fix quality**	Fix acquisition rate	Data transfer rate	Overall fix success rate	Deployment failure rate	Technical failure rate	Reference
2005	5	ST	---	south-east British Columbia, Canada	1	1	0	0	0	0	(15)
2006	14	ST/ AB	*Ursus arctos*	Montana, USA	1	1	0	0	0	0	(16)
2006	1	ST/ AB	*Ursus arctos*	southeast Finland	1	1	1	1	0	0	(17)
2007	6	ST/ AB	*Canis lupus, Cervus elaphus*	Alberta, Canada	0	1	1	1	1	1	(18)
2007	10	ST/ AB	*Ursus americanus*	Idaho, USA	1	1	0	0	0	0	(19)
2007	7	ST/ AB	*Ursus americanus*	Olympic National Park, Washington, USA	1	1	1	1	0	0	(20)
2007	1	ST/ AB	*Canis familiaris*	southwest France	1	1	0	0	0	0	(21)
2008	8	ST	---	Oregon, USA	1	1	0	0	0	0	(22)
2008	21	ST/ AB	*Ursus arctos*	British Columbia, Canada	0	1	0	0	0	0	(23)
2009	5	ST	---	Michigan, USA	1	1	0	0	0	0	(24)
2009	15	AB	*Ovis gmelini musimon x Ovis sp.*	France	0	1	0	0	1	0	(25)
2009	23	AB	*Odocoileus virginianus*	Minnesota, USA	0	1	0	0	1	1	(26)
2009	39	AB	*Ursus arctos; U. americanus*	Wyoming, USA	0	1	1	1	1	1	(27)
2010	42	ST/ AB	*Trichosurus vulpecula)*	Waikato, New Zealand	1	1	0	0	1	1	(28)
2010	9	ST/ AB	*Trichosurus vulpecula*	North Island, New Zealand	1	1	0	0	0	0	(29)
2010	104	AB	*Canis lupus, Equus hemonius, Equus ferus przewalski, Procapra gutturosa, Camelus ferus*	Mongolia	0	1	1	1	1	1	(30)
2010	48	AB	*Gulo gulo, Lynx lynx*	Northen Sweden	1	1	0	0	1	1	(31)

Year	# of deployments or collars*	Stationary/animal-borne	Species	Region	Fix quality**	Fix acquisition rate	Data transfer rate	Overall fix success rate	Deployment failure rate	Technical failure rate	Reference
2010	21	AB	*Cervus nippon*	Shiranuka Hills, Japan	1	1	0	0	1	1	(32)
2011	43	ST/ AB	*Felis catus*	South Island, New Zealand	1	1	0	0	0	0	(33)
2012	1	AB	*Papio hamadryas ursinus*	Western Cape, South Africa	1	1	0	0	1	1	(34)
2012	6	ST/ AB	*Lutra lutra*	Southern Portugal	1	1	1	1	1	1	(35)
2012	20	ST/ AB	*Cervus elaphus*	Germany	1	1	0	0	0	0	(36)
2012	11	ST	---	central New York State, USA	1	1	0	0	0	0	(37)
2013	3	ST	---	Dunedin, New Zealand	1	1	0	0	0	0	(38)
2013	18	ST/ AB	*Erinaceus europaeus*	UK	1	1	0	0	0	0	(39)
2013	4	AB	*Lynx lynx*	Dinaric alps	0	1	0	0	0	0	(40)
2013	280	AB	*Petrogale lateralis, Canis lupus dingo, Felis catus, Vulpes vulpes, Phascolarctos cinereus, Canis familiaris, Thylogale billardierii, Macropus rufogriseus rufogriseus, Trichosurus cunninghami, Dasyurus maculatus, Dasyurus geoffroii, Macropus giganteus, Vombatus ursinus*	Australia	1	1	1	1	0	1	(41)
2014	39	AB	*Urocyon littoralis*	Northwestern USA	1	1	1	1	1	1	(42)
2015	40	ST/ AB	*Canis familiaris*	Northeastern France	1	1	0	0	0	0	(43)
2015	59	AB	*Bison bison*	Yukon, Canada	1	1	0	0	1	1	(44)

Year	# of deployments or collars*	Stationary/animal-borne	Species	Region	Fix quality**	Fix acquisition rate	Data transfer rate	Overall fix success rate	Deployment failure rate	Technical failure rate	Reference
2015	56	ST/ AB	*Martes caurina*	California, USA	1	1	0	0	1	1	(45)
2016	35	AB	*Felis catus*	Australia	0	1	0	0	1	0	(46)
2017	23	ST/ AB	*Coragyps atratus; Cathartes aura*	Aiken, South Carolina, USA	1	1	0	0	1	1	(47)
2016	2	AB	*Tapirus terrestris*	Middle Caquetá river basin, Colombia	1	1	1	1	1	1	(48)
2016	1	AB	*Tayassu pecari*	Toledo, Belize	0	1	1	1	0	0	(49)
2017	30	ST/ AB	*Brachylagus idahoensis*	Lemhi Valley in east-central Idaho, USA	1	1	1	1	1	1	(50)
**Total**	**1228**				**40**	**50**	**15**	**15**	**23**	**22**	
**%**					**80**	**100**	**30**	**30**	**46**	**44**	

References

1. Rempel RS, Rodgers AR, Abraham KF. Performance of a GPS Animal Location System under Boreal Forest Canopy. J Wildl Manage. 1995;59(3):543–51.
2. Edenius L. Field test of a GPS location system for moose Alces alces under Scandinavian boreal conditions. Wildlife Biol. 1996;3:39–43.
3. Moen R, Pastor J, Cohen Y, Schwartz CC. Effects of moose movement and habitat use on GPS collar performance. J Wildl Manage. 1996;60(3):659–68.
4. Moen R, Pastor J, Cohen Y. Accuracy of GPS telemetry collar locations with differential correction. J Wildl Manage. 1997;61(2):530–9.
5. Bowman JL, Kochany CO, Demarais S, Leopold BD. Evaluation of a GPS collar for white-tailed deer. Wildl Soc Bull. 2000;28(1):141–5.
6. Biggs JR, Bennett KD, Fresquez PR. Relationship between home range characteristics and

the probability of obtaining successful global positioning system (GPS) collar positions for elk in New Mexico "Relationship between home range characteristics and the probability of obtaining succe. West North Am Nat. 2001;61(2).

7. Blake S, Douglas-Hamilton I, Karesh WB. GPS telemetry of forest elephants in Central Africa: Results of a preliminary study. Afr J Ecol. 2001;39(2):178–86.

8. Johnson CJ, Heard DC, Parker KL. Expectations and realities of GPS animal location collars: results of three years in the field. Wildlife Biol. 2002;8:153–9.

9. D'Eon R, Serrouya R, Smith G, Kochanny CO. GPS ratiotelemetry error and bias in mountainous terrain. Wildl Soc Bull. 2002;30(2):430–9.

10. Di Orio AP, Callas R, Schaefer RJ. Performance of Two GPS telemetry collars under different habitat conditions. Wildl Soc Bull. 2003;31(2):372–9.

11. Gau RJ, Mulders R, Ciarniello LM, Heard DC, Chetkiewicz C-LB, Boyce MS, et al. Uncontrolled field performance of Televilt GPS-Simplex ™ collars on grizzly bears in western and northern Canada. Wildl Soc Bull. 2004;32(3):693–701.

12. Sprague DS, Kabaya H, Hagihara K. Field testing a global positioning system (GPS) collar on a Japanese monkey: Reliability of automatic GPS positioning in a Japanese forest. Primates. 2004;45(2):151–4.

13. Janeau G, Adrados C, Joachim J, Gendner J-P, Pépin D. Performance of differential GPS collars in temperate mountain forest. C R Biol. 2004;327(12):1143–9.

14. Cain JW, Krausman PR, Jansen BD, Morgart JR. Influence of topography and GPS fix interval on GPS collar performance. Wildl Soc Bull. 2005;33(3):926–34.

15. D'Eon RG, Delparte D. Effects of radio-collar position and orientation on GPS radio-collar performance, and the implications of PDOP in data screening. J Appl Ecol. 2005;42(2):383–8.

16. Graves TA, Waller JS. Understanding the Causes of Missed Global Positioning System Telemetry Fixes. Wildl Soc Bull. 2006;70(3):844–51.

17. Sundell J, Kojola I, Hanski I. A New GPS–GSM-Based Method to Study Behavior of Brown Bears. Wildl Soc Bull. 2006;34(2):46–450.

18. Hebblewhite M, Percy M, Merrill EH. Are all global positioning system collars created equal? Correcting habitat-induced bias using three brands in the central Canadian Rockies. J Wildl Manage. 2007;71(6):2026–33.

19. Lewis JS, Rachlow JL, Garton EO, Vierling LA. Effects of habitat on GPS collar performance: using data screening to reduce location error. J Appl Ecol. 2007;44(3):663–71.

20. Sager-Fradkin KA, Jenkins KJ, Hoffman RA, Happe PJ, Beecham JJ, Wright RG. Fix success and accuracy of Global Positioning System collars in old-growth temperate coniferous forests. J Wildl Manage. 2007;71(4):1298–308.

21. Cargnelutti B, Coulon A, Hewison AJM, Goulard M, Angibault J-M, Morellet N. Testing Global Positioning System Performance for Wildlife Monitoring Using Mobile Collars and Known Reference Points. J Wildl Manage. 2007;71(4):1380–7.

22. Hansen MC, Riggs R a. Accuracy, precision, and observation rates of global positioning system telemetry collars. J Wildl Manage. 2008;72(2):518–26.

23. Heard DC, Ciarniello LM, Seip DR. Grizzly bear behavior and global positioning system collar fix rates. J Wildl Manage. 2008;72(3):596–602.

24. Belant JL. Effects of Antenna Orientation and Vegetation on Global Positioning System Telemetry Collar Performance. Northeast Nat. 2009;16(4):577–84.

25. Bourgoin G, Garel M, Dubray D, Maillard D, Gaillard J-M. What determines global positioning system fix success when monitoring free-ranging mouflon? Eur J Wildl Res.

2009;55(6):603–13.

26. Kochanny CO, Delgiudice GD, Fieberg J. Comparing Global Positioning System and Very High Frequency Telemetry Home Ranges of White-Tailed Deer. J Wildl Manage. 2009;73(5):779–87.

27. Schwartz CC, Podruzny S, Cain SL, Cherry S. Performance of Spread Spectrum Global Positioning System Collars on Grizzly and Black Bears. J Wildl Manage. 2009;73(7):1174–83.

28. Blackie HM. Comparative performance of three brands of lightweight Global Positioning System collars. J Wildl Manage. 2010;74(8):1911–6.

29. Dennis TE, Chen WC, Koefoed IM, Lacoursiere CJ, Walker MM, Laube P, et al. Performance Characteristics of Small Global-Positioning-System Tracking Collars for Terrestrial Animal. Wildl Biol Pract. 2010;6(1):14–31.

30. Kaczensky P, Ito TY, Walzer C. Satellite telemetry of large mammals in Mongolia: What expectations should we have for collar function? Wildl Biol Pract. 2010;6(2):108–26.

31. Mattisson J, Andrén H, Persson J, Segerström P. Effects of species behavior on global positioning system collar fix rates. J Wildl Manage. 2010;74(3):557–63.

32. Uno H, Suzuki T, Tachiki Y, Akamatsu R, Hirakawa H. Performance of GPS Collars Deployed on Free-Ranging Sika Deer in Eastern Hokkaido, Japan. Mammal Study. 2010;35(2):111–8.

33. Recio MR, Mathieu R, Denys P, Sirguey P, Seddon PJ. Lightweight GPS-tags, one giant leap for wildlife tracking? An assessment approach. PLoS One. 2011;6(12):e28225.

34. Pebsworth PA, Morgan HR, Huffman MA. Evaluating home range techniques: use of Global Positioning System (GPS) collar data from chacma baboons. Primates. 2012;53(4):345–55.

35. Quaglietta L, Martins BH, de Jongh A, Mira A, Boitani L. A Low-Cost GPS GSM/GPRS Telemetry System: Performance in Stationary Field Tests and Preliminary Data on Wild Otters (Lutra lutra). Clarke RH, editor. PLoS One. 2012;7(1):e29235. Available from: http://dx.plos.org/10.1371/journal.pone.0029235

36. Stache A, Löttker P, Heurich M. Red deer telemetry: Dependency of the position acquisition rate and accuracy of GPS collars on the structure of a temperate forest dominated by European beech and Norway spruce. Silva Gabreta. 2012;18(1):35–48.

37. Williams DM, Dechen Quinn A, Porter WF. Impact of Habitat-Specific GPS Positional Error on Detection of Movement Scales by First-Passage Time Analysis. Sueur C, editor. PLoS One. 2012;7(11):e48439.

38. Adams AL, Dickinson KJM, Robertson BC, van Heezik Y. An evaluation of the accuracy and performance of lightweight GPS collars in a suburban environment. PLoS One. 2013;8(7):e68496.

39. Glasby L, Yarnell RW. Evaluation of the performance and accuracy of Global Positioning System bug transmitters deployed on a small mammal. Eur J Wildl Res. 2013;59(6):915–9.

40. Krofel M, Skrbinšek T, Kos I. Use of GPS location clusters analysis to study predation, feeding, and maternal behavior of the Eurasian lynx. Ecol Res. 2013;28(1):103–16.

41. Matthews A, Ruykys L, Ellis B, Fitzgibbon S, Lunney D, Crowther MS, et al. The success of GPS collar deployments on mammals in Australia. Aust Mammal. 2013;35(1):65–83.

42. Cypher B, Drake E, Savage J, King J, Ralls K, Coonan T, et al. Evaluation of New Telemetry Technologies for Research on Island Foxes. Monogr West North Am Nat 7,. 2014;7:357–72.

43. Forin-Wiart M-A, Hubert P, Sirguey P, Poulle M. Performance and Accuracy of Lightweight and Low-Cost GPS Data Loggers According to Antenna Positions , Fix

Intervals , Habitats and Animal Movements. PLoS One. 2015;1–21.

44. Jung TS, Kuba K. Performance of GPS collars on free-ranging bison (*Bison bison*) in north-western Canada. Wildl Res. 2015;42:315–23.

45. Moriarty KM, Epps CW. Retained satellite information influences performance of GPS devices in a forested ecosystem. Wildl Soc Bull. 2015;39(2):349–57.

46. McGregor HW, Legge SM, Jones ME, Johnson CN. GPS collars are more efficient when collecting high-frequency data. Aust Mammal. 2016;38(2):237–40.

47. Byrne ME, Holland AE, Bryan AL, Beasley JC. Environmental conditions and animal behavior influence performance of solar-powered GPS-GSM transmitters. Condor. 2017;119(3):389–404.

48. Cabrera JA, Molina E, González T, Armenteras D. Does Plan B work ? Home range estimations from stored on board and transmitted data sets produced by GPS-telemetry in the Colombian Amazon. Int J Trop Biol. 2016;64(4):1441–50.

49. Hofman MPG, Signer J, Hayward MW, Balkenhol N. Spatial ecology of a herd of white-lipped peccaries (Tayassu pecari) in Belize using GPS telemetry : challenges and preliminary results. Therya. 2016;7(1):21–37.

50. McMahon LA, Rachlow JL, Shipley LA, Forbey JS, Johnson TR, Olsoy PJ. Evaluation of micro-GPS receivers for tracking small-bodied mammals. Crowther MS, editor. PLoS One.;12(3):e0173185.
