# Supplementary material for: Right on track? Performance of satellite telemetry in terrestrial wildlife research
Source: PLoS One. 2019 May 9;14(5):e0216223. doi: 10.1371/journal.pone.0216223 (PMC6508664; doi:10.1371/journal.pone.0216223)
Supplement: S2 Table — (PDF) [file pone.0216223.s011.pdf]

S2 Table. Tagged individuals per species. The number and relative proportion of individuals per species across all projects.

Species	Colloquial name	Number	Proportion
Alces alces	Moose	456	15.69%
Rangifer tarandus tarandus	Reindeer	311	10.70%
Ovis gmelini musimon x Ovis sp.	Mouflon sheep	239	8.22%
Acinonyx jubatus	Cheetah	169	5.81%
Capreolus capreolus	Roe deer	168	5.78%
Rupicapra rupicapra	Chamois	147	5.06%
Capra ibex	Alpine ibex	103	3.54%
Lepus europaeus	European hare	85	2.92%
Lynx lynx	Eurasian lynx	81	2.79%
Panthera pardus	Leopard	71	2.44%
Cervus elaphus	Red deer	70	2.41%
Ursus arctos	Brown bear	64	2.20%
Canis lupus dingo	Dingo	61	2.10%
Trichosurus vulpecula	Brushtail possum	58	2.00%
Phasianus colchicus	Ring-Necked Pheasant	55	1.89%
Ovis aries	Sheep	54	1.86%
Felis catus	Feral cat	49	1.69%
Brachylagus idahoensis	Pygmy rabbit	46	1.58%
Gulo gulo	Wolverine	43	1.48%
Panthera onca	Jaguar	43	1.48%
Sus scrofa	Wild boar	42	1.44%
Tayassu pecari	White-lipped peccary	42	1.44%
Equus hemonius	Asiatic wild ass	41	1.41%
Vulpes vulpes	Red fox	32	1.10%
Loxodonta africana	African elephant	28	0.96%
Panthera uncia	Snow leopard	25	0.86%
Caracal caracal	Caracal	22	0.76%
Papio papio	Guinea baboon	22	0.76%
Felis silvestris silvestris	European wildcat	21	0.72%
Phascolarctos cinereus	Koala	20	0.69%
Erinaceus europaeus	Hedgehog	18	0.62%

Species	Colloquial name	Number	Proportion
Alligator mississippiensis	American alligator	16	0.55%
Lepus americanus	Snowshoe hare	16	0.55%
Pekania pennanti	Fisher	16	0.55%
Parahyaena brunnea	Brown hyaena	15	0.52%
Urocyon littoralis	Island fox	14	0.48%
Erethizon dorsatum	North American porcupine	12	0.41%
Canis mesomelas	Black-backed jackal	10	0.34%
Crocuta crocuta	Spotted hyaena	10	0.34%
Meles meles	European badger	10	0.34%
Puma concolor	Puma	9	0.31%
Ursus americanus	American black bear	9	0.31%
Vulpes bengalensis	Indian fox	9	0.31%
Myrmecophaga tridactyla	Giant anteater	8	0.28%
Procyon lotor	Racoon	8	0.28%
Tragelaphus strepsiceros	Greater Kudu	7	0.24%
Connochaetes taurinus	Common wildebeest	6	0.21%
Leopardus colocolo	Pampas cat	5	0.17%
Leopardus jacobita	Andean cat	5	0.17%
Canis lupus familiaris	Domestic dog	4	0.14%
Eulemur rufifrons	Red-fronted lemur	4	0.14%
Felis chaus	Jungle cat	4	0.14%
Geochelone pardalis	Leopard tortoise	4	0.14%
Bos javanicus	Banteng	3	0.10%
Pantera leo	Lion	3	0.10%
Papio hamadryas	Hamadryas baboon	3	0.10%
Vulpes ferrilata	Tibetan fox	3	0.10%
Canis aureus	Golden jackal	2	0.07%
Otocolobus manul	Pallas cat	2	0.07%
Tapirus terrestris	South American tapir	2	0.07%
Panthera tigris	Tiger	1	0.03%
Tapirus bairdii	Central American tapir	1	0.03%
