# Supplementary material for: Right on track? Performance of satellite telemetry in terrestrial wildlife research
Source: PLoS One. 2019 May 9;14(5):e0216223. doi: 10.1371/journal.pone.0216223 (PMC6508664; doi:10.1371/journal.pone.0216223)

S1 Text. Trends in observed data.

Besides the identified effects in the boosted beta regressions, we detected a number of weak trends in the observed data and evaluated them using one-way ANOVA for the nominal variables, Pearson's correlation for ordinal variables and generalized linear models for continuous variables (see Table S1.1). However, note that the distribution of many variables did not comply with the normality assumption (even after transformation), and significance levels should be interpreted with caution. The main brand used in the project influenced all five metrics (Fig. S1.1 & S1.2). As species height decreased, the overall fix success rate was reduced as well, but primarily due to a decline in fix acquisition success (Fig. S1.3). The overall fix success rate decreased with increasing forest cover and density, mostly due to a decline in data transfer success, rather than in fix acquisition rate (Fig. S1.4, S1.5 & S1.6). Temperate and (sub)tropical evergreen forest had lower fix acquisition rates, which was reflected in the overall fix success rate (Fig. S1.7). The deployment failure rate decreased as units were more recently purchased (Fig. S1.8). Topography, unit price and the main means of transfer used in the project did not cause significant trends in any of the metrics.

Table S1.1. Observed data trends. Observed trends and significance levels for selected variables. (Significance levels: p < 0.001: ***; p < 0.01: **; p < 0.05: *; p > 0.05: -)

	Figure	Fix acquisition rate	Data transfer rate	Overall fix success rate	Deployment failure rate	Technical failure rate
**Brand**	1.1; 1.2	***	*	***	*	*
**Species height (log)**	S1.3	***	-	***	-	-
**Forest cover (quantitative)**	1.4	-	***	*	-	-
**Forest cover (qualitative)**	1.5	-	**	*	-	-
**Forest density**	1.6	-	**	*	-	-
**Forest type**	1.7	*	-	*	-	-
**Purchase date**	1.8	-	-	-	**	-
**Data transfer method**	-	-	-	-	-	-
**Terrain ruggedness**	-	-	-	-	-	-
**Terrain Ruggedness Index**	-	-	-	-	-	-
**Unit price**	-	-	-	-	-	-

Fig. S1.1. Observed success rates per unit brand. Observed success rates summarized per brand (i.e. the brand of the majority of units in each study). Comparison among brands was not conducted due to unequal sample sizes, but the effect of the brand was significant for all three metrics (see Table S1.1; Significance levels: $p < 0.001$: ***; $p < 0.01$: **; $p < 0.05$: *).

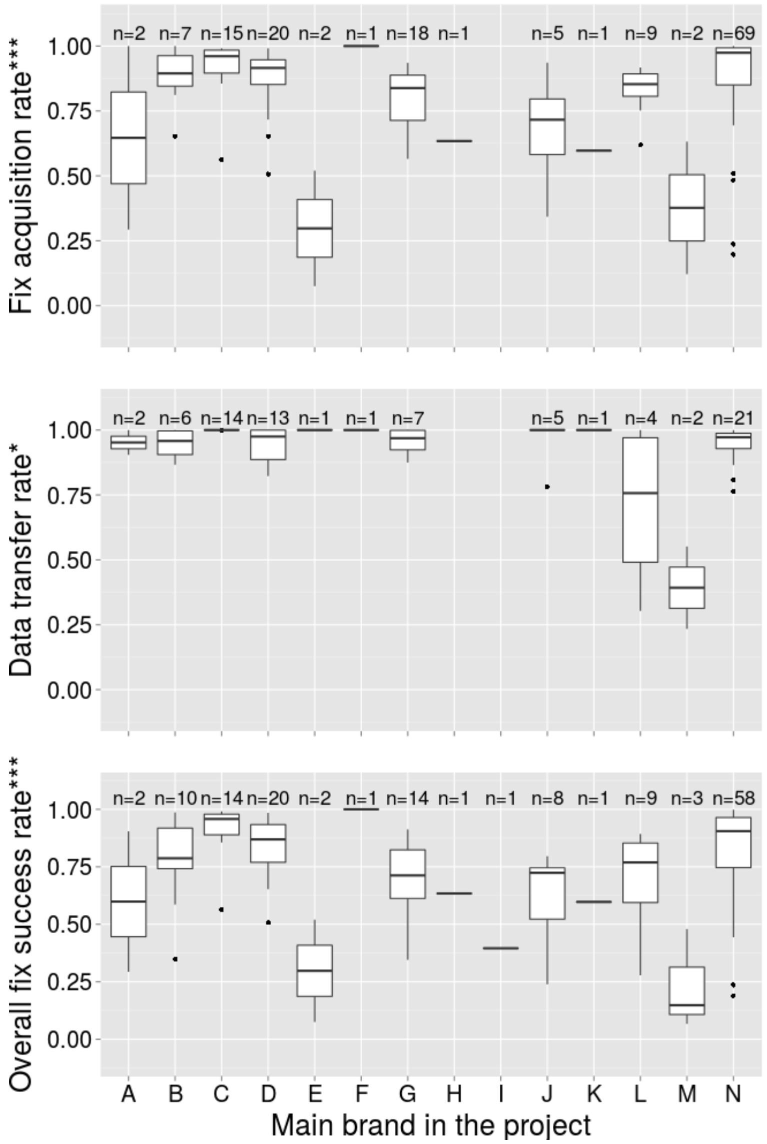

Fig. S1.2. Observed failure rates per unit brand. Observed failure rates summarized per brand (i.e. the brand of the majority of units in each study). Comparison among brands was not conducted due to unequal sample sizes, but the overall effect of the brand was significant for all two metrics (see Table S1.1; Significance levels: $p < 0.001$: ***; $p < 0.01$: **; $p < 0.05$: *).

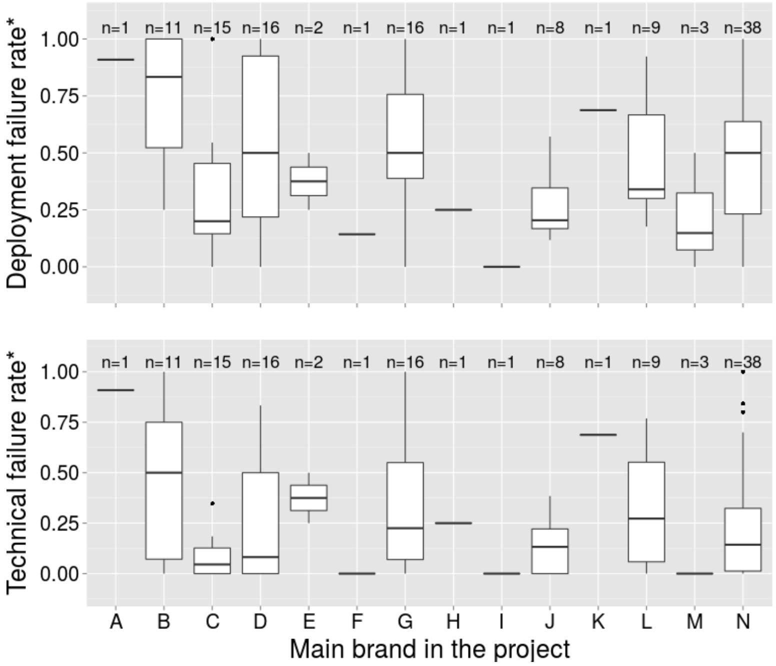

Fig. S1.3. Observed success rates against body height. Observed success rates plotted against the weighted mean of height across all individuals in a study. The trend was significant for the fix acquisition rate and the overall fix success rate metrics (see Table S1.1; Significance levels: $p < 0.001$: ***; $p < 0.01$: **; $p < 0.05$: *).

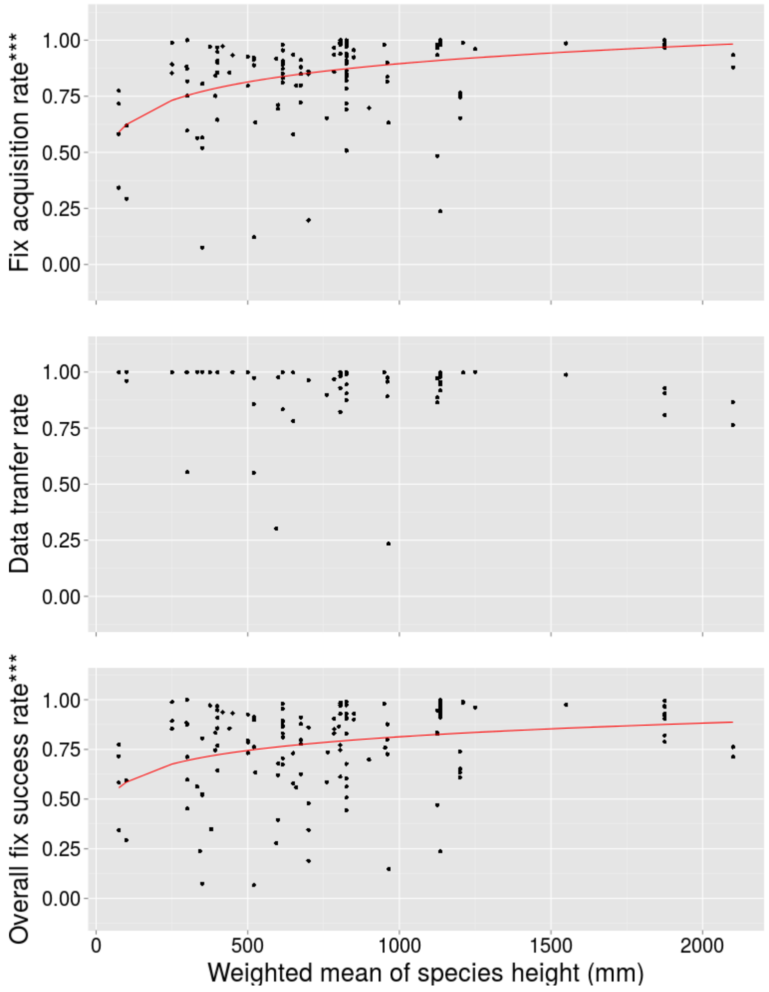

Fig. S1.4. Observed success rates against forest cover (quantitative). Observed success rates plotted against the percentage forest cover in the projects' study area, according to the GlobCover dataset. Both the data transfer rate and the overall fix success rate decreased with increasing forest cover (see Table S1.1; Significance levels: $p < 0.001$: ***; $p < 0.01$: **; $p < 0.05$: *).

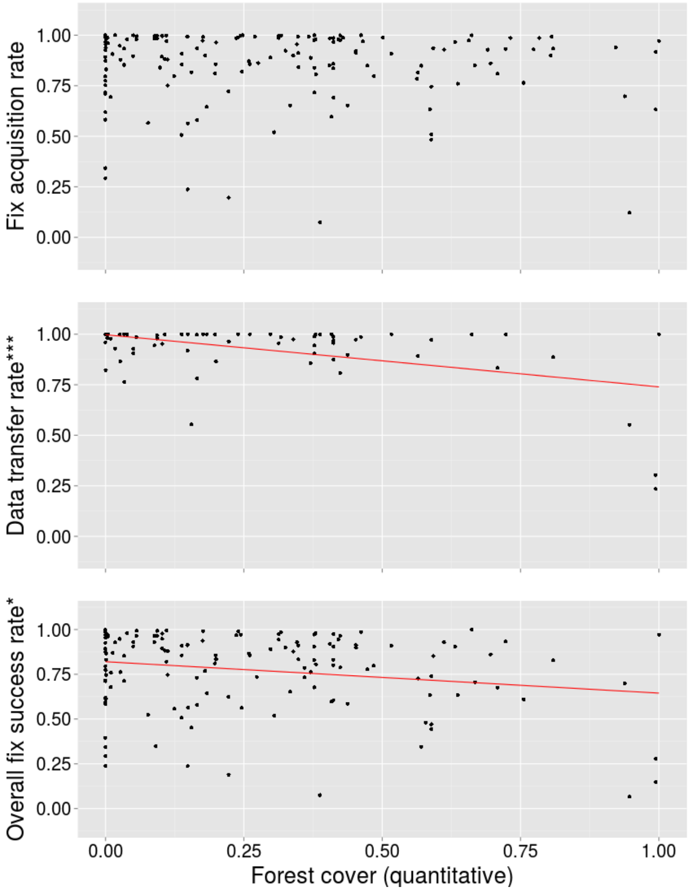

Fig. S1.5. Observed success rates against forest cover (qualitative). Observed success rates plotted against the percentage forest cover in the projects' study area, according to the information provided in the questionnaire. As with the quantitative forest cover (Figure S1.4), both the data transfer rate and the overall fix success rate decreased with increasing forest cover (see Table S1.1; Significance levels: $p < 0.001$: ***; $p < 0.01$: **; $p < 0.05$: *).

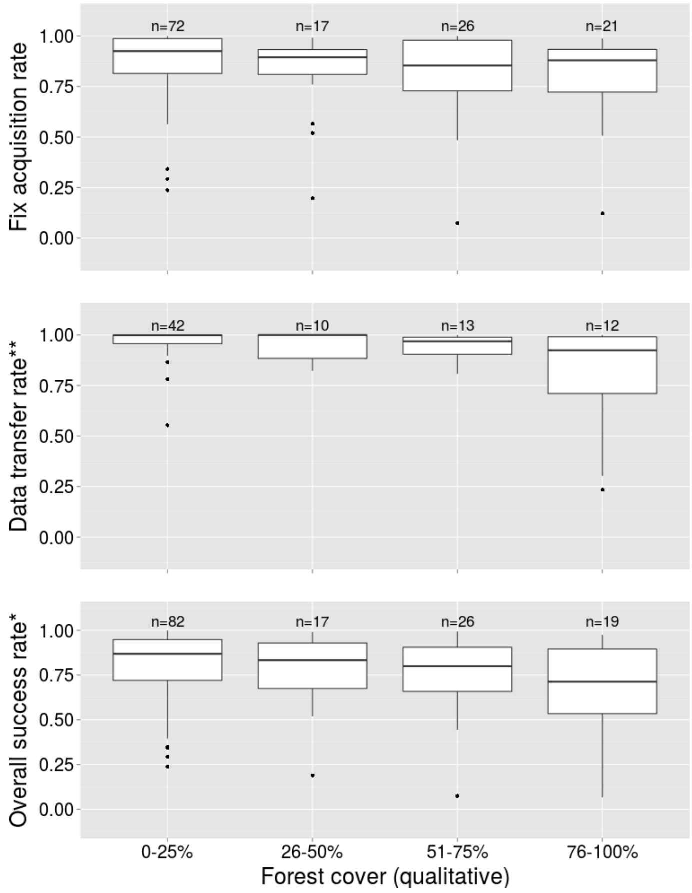

Fig. S1.6. Observed success rates against forest density. Observed success rates plotted against the forest density in the projects' study area, according to the information provided in the questionnaire. 0 = No forest; 1 = Open understory, sparse canopy cover; 2 = Dense understory, sparse canopy cover; 3 = Open understory, intermediate canopy cover; 4 = Dense understory, intermediate canopy cover; 5 = Open understory, closed canopy; 6 = Dense understory, closed canopy. Both the data transfer rate and the overall fix success rate decreased with increasing forest density (see Table S1.1; Significance levels: $p < 0.001$: ***; $p < 0.01$: **; $p < 0.05$: *). The rather low fix acquisition rate and overall fix success rate under 'No forest' conditions are likely due to the fact that the majority of project areas without forest cover were mountainous areas where topography may be expected to reduce success rates.

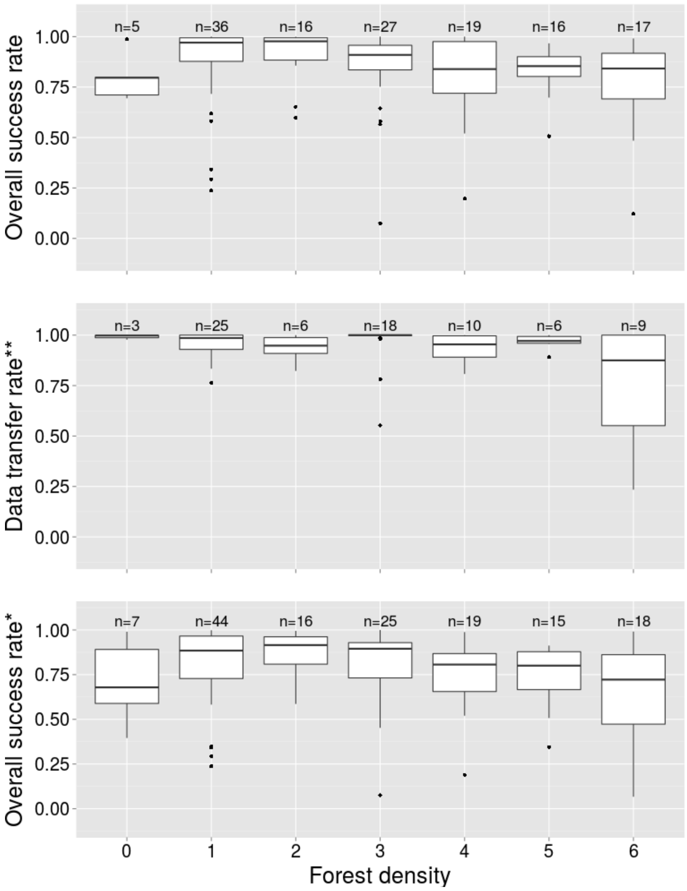

Fig. S1.7. Observed success rates against forest type. Observed success rates plotted against the type of forest in the projects' study area. 0 = No forest; 1 = Temperate evergreen; 2 = Temperate deciduous; 3 = Temperate mixed; 4 = (Sub)Tropical evergreen; 5 = (Sub)Tropical deciduous; 6 = (Sub)Tropical mixed. Both the fix acquisition rate and the overall fix success rate were influenced by forest type, with the evergreen forests generally having lower success rates (see Table S1.1; Significance levels: $p < 0.001$: ***; $p < 0.01$: **; $p < 0.05$: *). Similar to Fig. S1.6, the rather low fix acquisition rate and overall fix success rate under 'No forest' conditions are likely due to the fact that the majority of project areas without forest cover were mountainous areas where topography may be expected to reduce success rates.

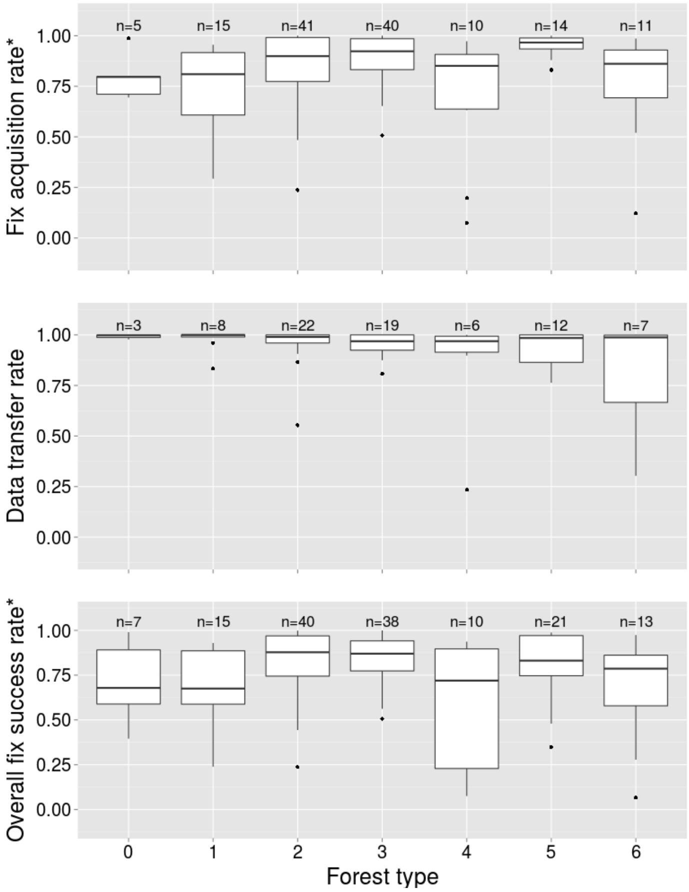

Fig. S1.8. Failure rates per unit year of purchase. Deployment and technical failure rates plotted against the weighted mean purchase year across all units in a study. More recent units had a lower deployment failure rate. (Significance levels: $p < 0.001$: ***; $p < 0.01$: **; $p < 0.05$: *).

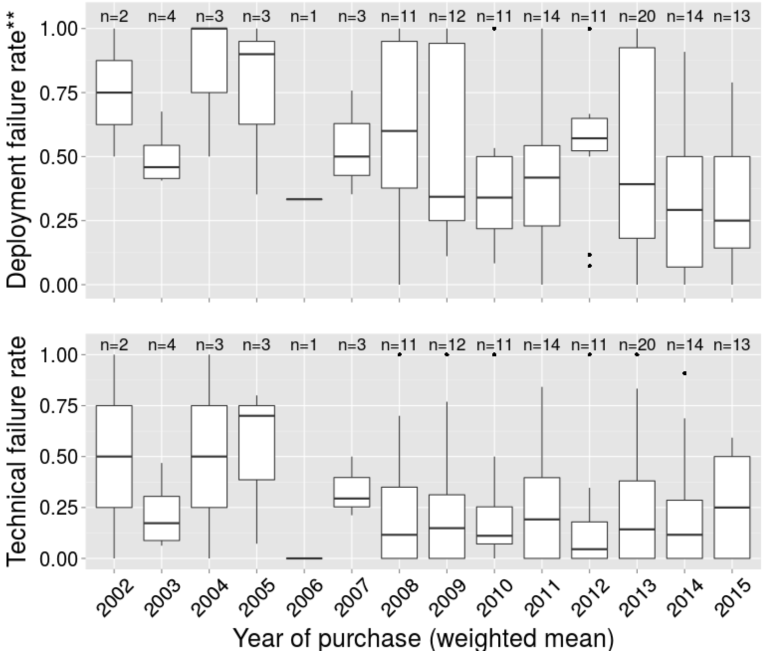

Supplement: S1 Text — (PDF) [file pone.0216223.s012.pdf]
