# Supplementary material for: Right on track? Performance of satellite telemetry in terrestrial wildlife research
Source: PLoS One. 2019 May 9;14(5):e0216223. doi: 10.1371/journal.pone.0216223 (PMC6508664; doi:10.1371/journal.pone.0216223)
Supplement: S2 Text — (PDF) [file pone.0216223.s013.pdf]

S2 Text. Unit purchase and operation costs.

The purchase costs of units were between US$2,001-2,500 in 39.7% of the projects, US$2,000 or cheaper in 32.9%, and more expensive than US$2,500 in 27.4%. In over half of the projects (52.3%), the additional costs for software licenses and/or hardware extensions were US$50 or less, however in 19.6% of the projects these costs exceeded US$500. Units with GSM or satellite data transfer tended to be more expensive to purchase and have higher recurring costs. Monthly costs for GSM contracts or satellite services can accumulate fast. In 57.2% of all projects, these monthly costs remained below US$10 per unit, while in 14.5% these costs were more than US$50. Purchase and operating costs for units with satellite and GSM data transfer are usually higher than for VHF/UHF or store on board units. However, the overall operating costs for VHF/UHF transfer and store-on-board systems are difficult to calculate due to the increased field effort required to retrieve the data and/or units [1]. A better alternative for estimating cost efficiency of wildlife satellite telemetry is to calculate the cost per fix [1,2]. However, because our data collection and analysis was on the project level, we could not calculate a cost per fix for the average collar. Therefore, we were not able to estimate the cost efficiency of satellite telemetry devices.

Researcher opinion on data usefulness

Most researchers considered a fix acquisition rate of around 90% or more to be good, while anything lower than 80% was regarded poor. However, an overall fix success rate of more than 80% was considered good.

References

1. Thomas B, Holland JD, Minot EO. Wildlife tracking technology options and cost considerations. Wildl Res. 2011;38: 653–663. Available: http://dx.doi.org/10.1071/WR10211

2. Matthews A, Ruykys L, Ellis B, Fitzgibbon S, Lunney D, Crowther MS, et al. The success of GPS collar deployments on mammals in Australia. Aust Mammal. 2013;35: 65–83. doi:10.1071/AM12021
